# Deep self-learning enables fast, high-fidelity isotropic resolution restoration for volumetric fluorescence microscopy

**DOI:** 10.1038/s41377-023-01230-2

**Published:** 2023-08-28

**Authors:** Kefu Ning, Bolin Lu, Xiaojun Wang, Xiaoyu Zhang, Shuo Nie, Tao Jiang, Anan Li, Guoqing Fan, Xiaofeng Wang, Qingming Luo, Hui Gong, Jing Yuan

**Affiliations:** 1 Britton Chance Center for Biomedical Photonics, Wuhan National Laboratory for Optoelectronics, Huazhong University of Science and Technology, Wuhan, China; 2 MoE Key Laboratory for Biomedical Photonics, School of Engineering Sciences, Huazhong University of Science and Technology, Wuhan, China; 3 HUST-Suzhou Institute for Brainsmatics, Suzhou, China; 4 School of Biomedical Engineering, Hainan University, Haikou, China

**Keywords:** Microscopy, Biophotonics

## Abstract

One intrinsic yet critical issue that troubles the field of fluorescence microscopy ever since its introduction is the unmatched resolution in the lateral and axial directions (i.e., resolution anisotropy), which severely deteriorates the quality, reconstruction, and analysis of 3D volume images. By leveraging the natural anisotropy, we present a deep self-learning method termed Self-Net that significantly improves the resolution of axial images by using the lateral images from the same raw dataset as rational targets. By incorporating unsupervised learning for realistic anisotropic degradation and supervised learning for high-fidelity isotropic recovery, our method can effectively suppress the hallucination with substantially enhanced image quality compared to previously reported methods. In the experiments, we show that Self-Net can reconstruct high-fidelity isotropic 3D images from organelle to tissue levels via raw images from various microscopy platforms, e.g., wide-field, laser-scanning, or super-resolution microscopy. For the first time, Self-Net enables isotropic whole-brain imaging at a voxel resolution of 0.2 × 0.2 × 0.2 μm^3^, which addresses the last-mile problem of data quality in single-neuron morphology visualization and reconstruction with minimal effort and cost. Overall, Self-Net is a promising approach to overcoming the inherent resolution anisotropy for all classes of 3D fluorescence microscopy.

## Abstract (web summary)

We propose a deep self-learning approach driven by optical principles for fast and high-fidelity 3D isotropic resolution restoration for volumetric microscopy.

## Graphical abstract

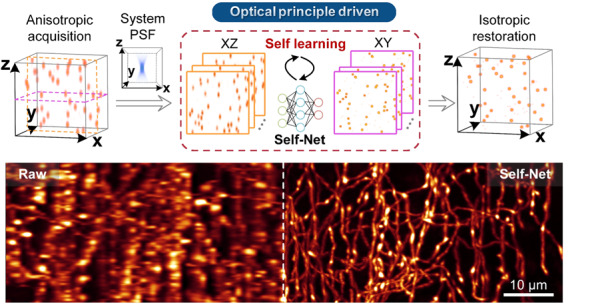

## Introduction

Volumetric fluorescence microscopy is an indispensable tool for comprehensive studies of cells and organs. Since the specimens are inherently three-dimensional (3D), the optimal imaging system should possess high spatial resolution in all directions^1,2^. However, limited by the operation principle, most microscopic imaging modalities suffer from an anisotropic point spread function (PSF)^3^, i.e., the axial resolution is worse than the lateral resolution by two to three times, which severely hinders the accurate visualization as well as dissection and analysis of complex volumetric structures inside the biological samples. For example, the resolution anisotropy causes great challenges in reconstructing complex single-neuron morphologies for high-resolution (HR) whole-brain imaging^4–6^ owing to the inability to accurately distinguish individual neurites and judge their connections in densely-packed regions^7^. As the multiple neurites of different neurons, sometimes of a single neuron, frequently interweave in 3D local space, it is highly desired to observe such data from multiple perspectives for accurate reconstruction. However, the poor Z-resolution of the raw data restricts such observability, leading to a high frequency of false links. The high error rate in local complex areas could finally lead to severe reconstruction errors of the whole neuron morphology, making the subsequent analyses unreliable.

Although some advanced hardware modalities allow 3D imaging at an isotropic resolution^8–14^, these approaches are relatively sophisticated, restricting their wide applicability. As a leading data-driven approach, deep learning may provide an attractive computational alternative. The key to this scheme is to solve the 3D anisotropy of the non-spherical optical PSF. Recently, deep learning has been demonstrated to be very effective and applicable in overcoming other inherent drawbacks and alleviating performance tradeoffs in fluorescence microscopy^15–23^. The common strategy for training deep neural networks is supervised learning on a large set of well-registered training pairs. However, for isotropic recovery, it is extremely difficult to acquire precisely-matched training data with routine microscopes. Although training data can be generated semi-synthetically, e.g., content-aware image restoration (CARE)^24^, this approach requires the estimation of the system PSF and the performance highly depends on the accuracy of the physical model for generating paired training data. Recently, the invention of a cycle-consistent generative adversarial network (CycleGAN) has made it possible to train neural networks using unpaired data^25^. A newly reported work has employed the 3D optimal transport-driven CycleGAN network (OT-CycleGAN) for isotropic recovery of several volumetric imaging data^26^. However, training this big 3D network is not trivial. It requires large memory consumption and a long time for training and inference. More importantly, the problem of anisotropy correction is specific as it requires high-fidelity output but not a simple style transfer. Therefore, only using the weak constraint introduced by cycle consistency is prone to generate noise artifacts and structural distortion in the output images when directly learning the image transformation from the anisotropic domain to the isotropic domain, bringing risks to real biomedical applications^27^.

Here, we present a general-purpose two-stage deep self-learning approach termed Self-Net to improve the resolution isotropy for volumetric fluorescence microscopy with fast training and inference speed as well as high reconstruction fidelity. Our approach employs the self-learning strategy that improves the axial resolution of the anisotropic raw data by using the HR lateral images in the same dataset as rational targets. This strategy fully exploits the 3D distribution characteristics of the system PSF and eliminates the need for acquiring registered training data or physically modeling the image formation process. Distinct from the previous approaches, Self-Net employs unsupervised learning for realistic anisotropic degradation, thus allowing the construction of supervised training to impose a strong constraint on the isotropic restoration results, to ensure high-fidelity reconstruction. We validate the reliability and effectiveness of Self-Net using both simulated and experimentally acquired data. We demonstrate that Self-Net overcomes the resolution anisotropy of wide-field, optical-sectioning, and super-resolution microscopies through volumetric imaging of diverse samples. Furthermore, the high effectiveness and applicability of Self-Net enable a time- and data-efficient pipeline to achieve isotropic whole-brain imaging (DeepIsoBrain) at a voxel resolution of 0.2 × 0.2 × 0.2 μm^3^, which significantly improves the reconstruction efficiency and accuracy of complex single-neuron morphology and can be a promising approach to facilitate morphology-based neuroscience research.

## Results

### Two-stage self-learning strategy and performance validation of Self-Net

**Fig. 1:**
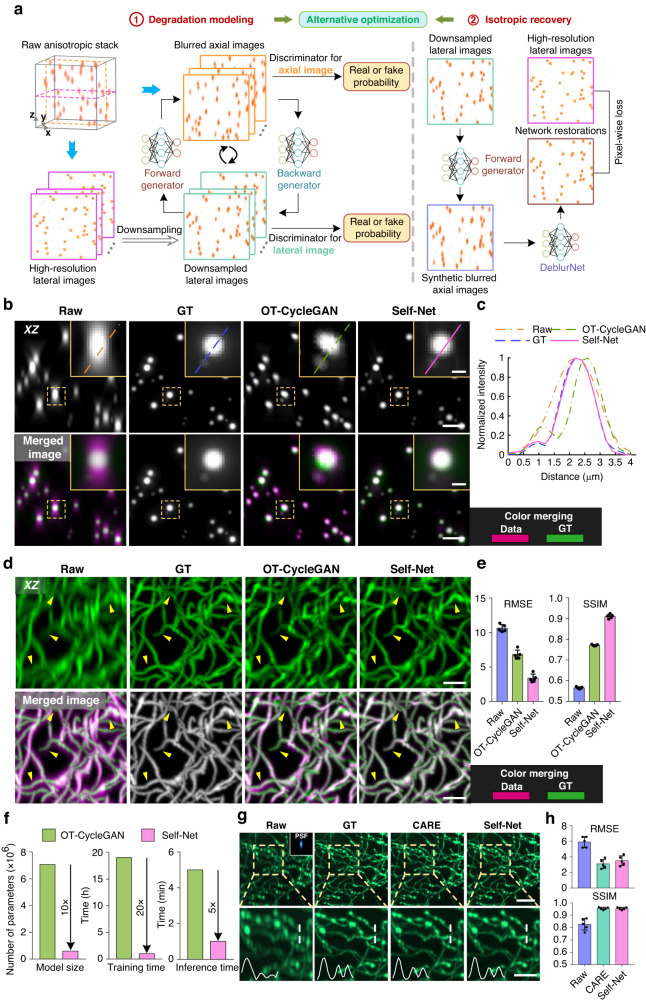
Self-Net pipeline and validation of its isotropic recovery performance using synthetic and semisynthetic data. **a** Self-learning strategy and schematic of Self-Net. **b** First row: XZ maximum-intensity projections (MIPs) of the raw anisotropic data, isotropic ground truth (GT) data, OT-CycleGAN, and Self-Net restorations of simulated fluorescence beads. The projection thickness is 50 μm. Second row: corresponding color-merged images by merging the data (magenta) and GT (green). The insets show enlarged views of the corresponding dashed boxes. Scale bar: 5 μm; 1 μm for the insets. **c** Intensity profiles along the corresponding colored lines in the insets in (**b**). **d** First row: XZ MIPs of the raw anisotropic data, isotropic GT data, OT-CycleGAN, and Self-Net restorations of synthetic tubular volume. The projection thickness is 30 slices. The yellow arrowheads highlight the details inconsistent with the GT data in the OT-CycleGAN output. Second row: corresponding color-merged images by merging the data (magenta) and GT (green). Scale bar: 20 pixels. **e** Quantitative comparison of the image qualities of d via the RMSE and the SSIM. Results are obtained from five randomly-selected MIP images of 900 × 900 pixels. **f** Comparison of model size, training time, and inference time between the OT-CycleGAN and Self-Net using the same GPU card (GeForce RTX 3090, Nvidia). The inference time was evaluated using a 700^3^ voxels volume. **g** MIPs of the raw image stack (top right: PSF kernel used to generate anisotropic blurring), GT image stack, CARE restoration (supervised learning), and Self-Net restoration (unsupervised learning) of semisynthetic neuronal data. The projection thickness is 300 μm. Normalized intensity profiles along the corresponding white dashed lines in the enlarged images are shown in the bottom-left corner of the images. Scale bar: 10 μm; 5 μm for the enlarged images. **h** Quantitative comparison of the image qualities of g via RMSE and SSIM (*n* = 5 independent image stacks)

Figure 1a illustrates the two-stage self-learning strategy of Self-Net. Instead of viewing each 3D data as an entirety, we sliced it into 2D image sets containing multiple lateral and axial planes. The HR lateral images naturally served as the rational gold standard for enhancing the axial resolution of the raw data due to the anisotropy of the optical PSF. Therefore, we directly utilized unpaired lateral and axial images retrieved from the same anisotropic volume to learn the axial-to-lateral mappings through unsupervised training. In this way, a single raw image stack is sufficient for network training. To overcome the hallucination problem owing to the weak constraint in the previous CycleGAN-based unsupervised approach, we employed unsupervised training to learn transformation from the HR lateral images to the blurred axial images instead of directly reconstructing HR images as did in previous approaches. This is based on our finding that in unpaired settings, networks learn image degradation better and are more stable than image deblurring (Fig. S1). With the help of realistic anisotropic PSF blurring, we were able to construct supervised training to impose a strong constraint on the isotropic restoration results, thereby ensuring the output was unaffected by hallucinatory features. The workflow of Self-Net is as follows: In the first stage, unpaired lateral and axial slices obtained from the same image stack were fed into the CycleGAN model to learn the anisotropic degradation process. Due to the lower sampling rate in the axial direction (than in the lateral directions), we downsampled the lateral images to reduce the resolution gap between the lateral and axial views. This process also helped the networks focus on learning the anisotropic PSF. In the second stage, the blurred axial images generated by the first-stage network combined with pixel-aligned HR lateral images were used to train the isotropic recovery network (named DeblurNet) through pixel-wise reconstruction loss. Finally, we established feedback between these two stages so that the training loss of DeblurNet can guide the first-stage module to generate more realistic blurred images. We alternately optimized the two stages until both converge. In the testing phase, isotropic restoration was realized by loading the DeblurNet to enhance all axial slices in the original image stack (Fig. S2).

We verified the isotropic recovery performance of Self-Net using simulated fluorescence beads. We used their image stack as ground truth (GT) data and convolved them with a theoretical wide-field 3D PSF that exhibited a lateral full-width-at-half-maximum (FWHM) of 305 nm and an axial FWHM of 1000 nm. For comparison, we trained Self-Net and OT-CycleGAN using this anisotropic data (Fig. 1b, c). The axial elongation of the PSF severely distorted objects and caused weak signals to be obscured in the raw data. The output of the OT-CycleGAN network exhibited improved axial resolution, but compared with GT, the restored beads still had obvious deviations in shape and intensity. In contrast, our Self-Net effectively removed the axial blur from the raw image stack and provided an isotropic restoration consistent with the GT data (Supplementary Video 1).

We also generated and tested synthetic tubular volumes for performance validation, as did in ref. ^26^ (Fig. 1d; Methods). The OT-CycleGAN produced visually-enhanced output, but many details were inconsistent with the GT (as highlighted by yellow arrowheads in Fig. 1d). Our Self-Net generated high-fidelity reconstructions that matched well with the GT data. For quantitative comparison, we measured the root-mean-square error (RMSE) and the structural similarity index (SSIM) between the restored images and the GT (Fig. 1e). The results showed that Self-Net yielded notably higher image quality metrics than OT-CycleGAN. Moreover, compared to large 3D networks of OT-CycleGAN, our Self-Net has 10× fewer parameters and is 20× and 5× faster in training and inference time, respectively (Fig. 1f), which indicates that our Self-Net is fast, compact, and easily optimizable.

We next compared the performance of Self-Net under supervised and unsupervised settings using semisynthetic image data (Fig. 1g and S3; Methods). Note that, the supervised training can be regarded as the replication of the CARE method^24^ under the ideal condition (i.e., the lateral-to-axial degradation process is accurately modeled). We show that both networks provided resolution-enhanced images that suitably matched the GT data. The comparable RMSE and SSIM metrics of Self-Net with CARE (Fig. 1h) demonstrated that the network still learned to perform effective anisotropy correction even without paired data. We also evaluated the effect of different extents of resolution anisotropy on the isotropic recovery performance of the networks (Fig. S4). The results showed that in all cases, both CARE and Self-Net had considerable resolution improvement compared to the input. And like all deep-learning methods, the performance of Self-Net degraded at increasing anisotropy and when the gap in lateral and axial resolution is greater than 4 times, the deterioration became particularly obvious.

### Self-Net improves resolution isotropy in 3D imaging of biological tissues

**Fig. 2:**
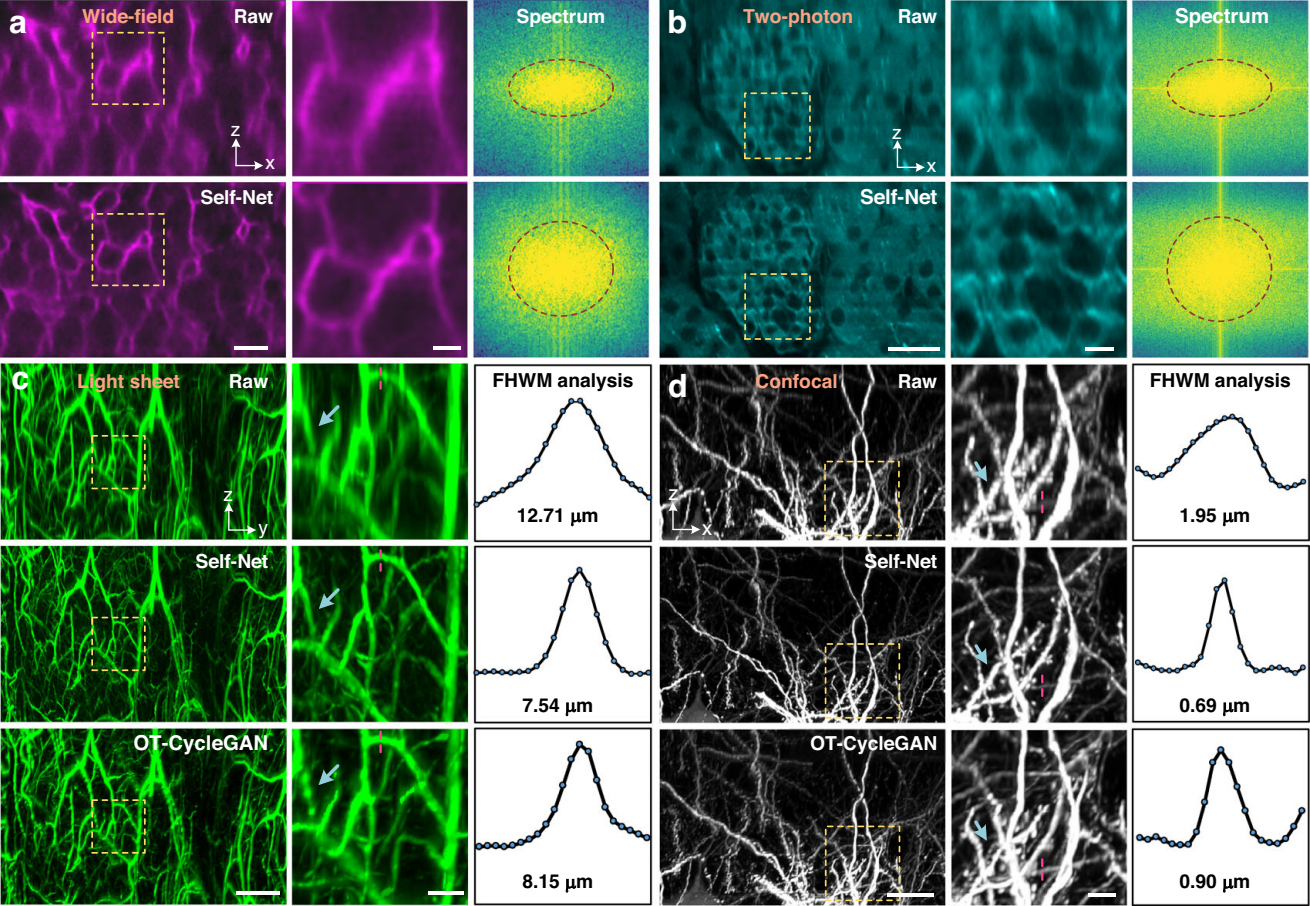
Demonstration of Self-Net for 3D isotropic imaging of various tissues. Wide-field and two-photon imaging of cleared mTmG mouse liver (**a**) and kidney tissues (**b**), respectively. From left to right, the images, enlarged views of the corresponding dashed boxes in the images, and the Fourier spectrum of the images are exhibited. The raw images and Self-Net restorations are shown in the first and second rows, respectively. Scale bar: 25 μm; 5 μm for the enlarged images. Light-sheet and confocal imaging of cleared mouse brain vasculature (**c**) and neurons (**d**), respectively. From left to right, the images, enlarged views of the corresponding dashed boxes in the images, and the intensity profiles along the corresponding red dashed lines are exhibited. The raw images and their Self-Net and OT-CycleGAN restorations are shown in turn in three rows. The blue arrows indicate that the structural information in the raw axial images was inconsistent with the OT-CycleGAN output, while consistent with the Self-Net output. Scale bar: 200 μm (**c**) and 20 μm (**d**); 50 and 5 μm for the corresponding enlarged images in (**c**) and (**d**), respectively

We verified the isotropic restoration performance of our Self-Net on real biological data acquired by commercial wide-field, two-photon, confocal, and light-sheet microscopes. Detailed information on all the training data is available in Supplementary Table 1. The images of the mouse liver (Fig. 2a), kidney (Fig. 2b), brain vessels (Fig. 2c, raw data were released by ref. ^28^), and neurons (Fig. 2d) all exhibited significant improvement in the axial resolution after Self-Net restoration (Supplementary Video 2). Fine structures such as microvascular architecture, glomerular tufts, and dendrite spines can now be visualized in the axial views. We also made a comparison with the OT-CycleGAN network and showed that our Self-Net had obvious improvement in restoration quality. To intuitively demonstrate the resolution enhancement, we performed Fourier spectrum analysis and FWHM analysis (please also refer to Fig. S5) and quantified the isotropy improvement of each data (Fig. S6 and Supplementary Table 2). Moreover, we demonstrated that Self-Net can be applied to imaging data with signal attenuation and non-uniform PSF blurring with depth (Fig. S7). These results demonstrated that Self-Net has the capability to improve the resolution isotropy of various microscopy and help better observe and analyze 3D morphologies of tissues and organs from any perspective.

### Application of Self-Net in super-resolution volumetric microscopy

**Fig. 3:**
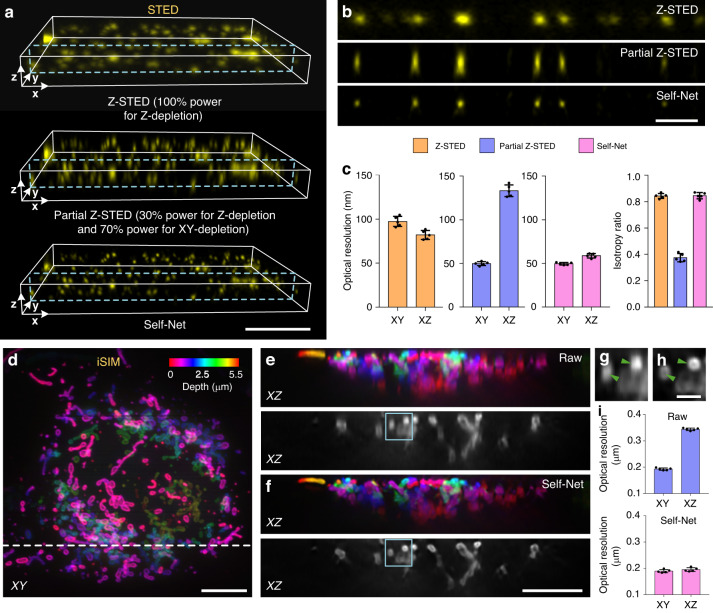
Isotropic restoration of super-resolution imaging data. **a** Volume rendering of the Z-STED (100% depletion power for Z), partial Z-STED imaging data, and Self-Net output of nanobeads. Scale bar: 1 μm. **b** XZ cross-sections indicated by the blue dashed boxes in a. Scale bar: 0.5 μm. **c** 3D resolution quantification of the data in (**a**). **d** Color-coded XY MIP of the raw iSIM data after deconvolution. Scale bar: 5 μm. **e**, **f** Color-coded XZ MIPs and their typical cross-sections indicated by the white dotted line in (**d**) of the raw data (**e**) and Self-Net-restored data (**f**). Scale bar: 5 μm. **g**, **h** Enlarged views of the corresponding regions indicated by the blue box in (**e**) and (**f**), respectively. The green arrowheads highlight the hollow structures of mitochondria, which were blurry in the original axial view and resolved in the Self-Net reconstructed image. Scale bar, 1 μm. **i** Optical resolutions of the raw data and Self-Net-restored data by measuring the FWHMs of sub-diffractive objects in the corresponding image volumes

Achieving high isotropic resolution beyond the diffraction limit is still technically challenging at present. Here, we demonstrated the unique capability of Self-Net to tackle this problem. For example, although the 3D stimulated emission depletion (STED) microscopy^29^ can provide subdiffraction Z-resolution by superimposing a ‘bottle-shaped’ beam, the finite budget of laser power for 3D depletion leads to a performance tradeoff between the lateral and axial resolutions. To demonstrate this, we imaged fluorescent nanobeads in 3D using a commercial STED system (Abberior Facility Line; Fig. 3a–c). To enhance the signal-to-noise ratio, the imaged data were deconvolved using the Richardson–Lucy (RL) algorithm^30,31^ with iterations of 5. We used the mode of 100% Z-depletion power to achieve Z-STED imaging and obtained the lateral and axial resolutions of 97.3 ± 5.7 nm (mean ± standard deviation) and 82.0 ± 5.1 nm, respectively. Then, we used 70% XY-depletion power and 30% Z-depletion power to obtain a relatively high lateral resolution of 49.8 ± 2.3 nm by sacrificing the axial resolution to 133.2 ± 6.7 nm. We recovered this STED data to nearly isotropic resolution (49.6 ± 1.4 nm laterally and 58.7 ± 2.5 nm axially) by Self-Net processing and showed twofold lateral and 1.4-fold axial resolution improvements compared to conventional Z-STED imaging (Supplementary Video 3). This result confirms that Self-Net can push the limit of 3D STED with limited depletion power.

As another representative super-resolution microscopy, 3D structured illumination microscopy (SIM) also suffers from resolution anisotropy^32^ (very few modified techniques allow for isotropic acquisitions^33,34^). To test the ability of Self-Net to overcome this defect, we used previously released instant SIM (iSIM)^35^ imaging data of fixed U2OS cells transfected with mEmerald-Tomm20 labeling outer mitochondrial membrane^21^. In the raw data after deconvolution, mitochondria can be visualized in the lateral views (Fig. 3d), but still were ambiguous in the axial views (Fig. 3e). After Self-Net restoration, the fine structures of individual mitochondrion became super-resolved in the axial direction (Fig. 3f and Supplementary Video 4). The hollow structures of mitochondria (indicated by the green arrowheads) that were blurry in the original axial view (Fig. 3g) were resolved in the Self-Net reconstructed image (Fig. 3h). We performed the FWHM analysis of sub-diffractive objects in the images to quantify the 3D optical resolutions and showed that Self-Net improved the axial resolution from 344 ± 5 to 195 ± 7 nm, matching the lateral resolution (189 ± 6 nm; Fig. 3i). These results highlighted the potential of Self-Net to enable in live-cell 3D isotropic imaging and facilitate 3D visualization and analysis of intracellular structures.

### Self-Net enables fast and accurate morphology reconstruction of densely-interweaving neurites

**Fig. 4:**
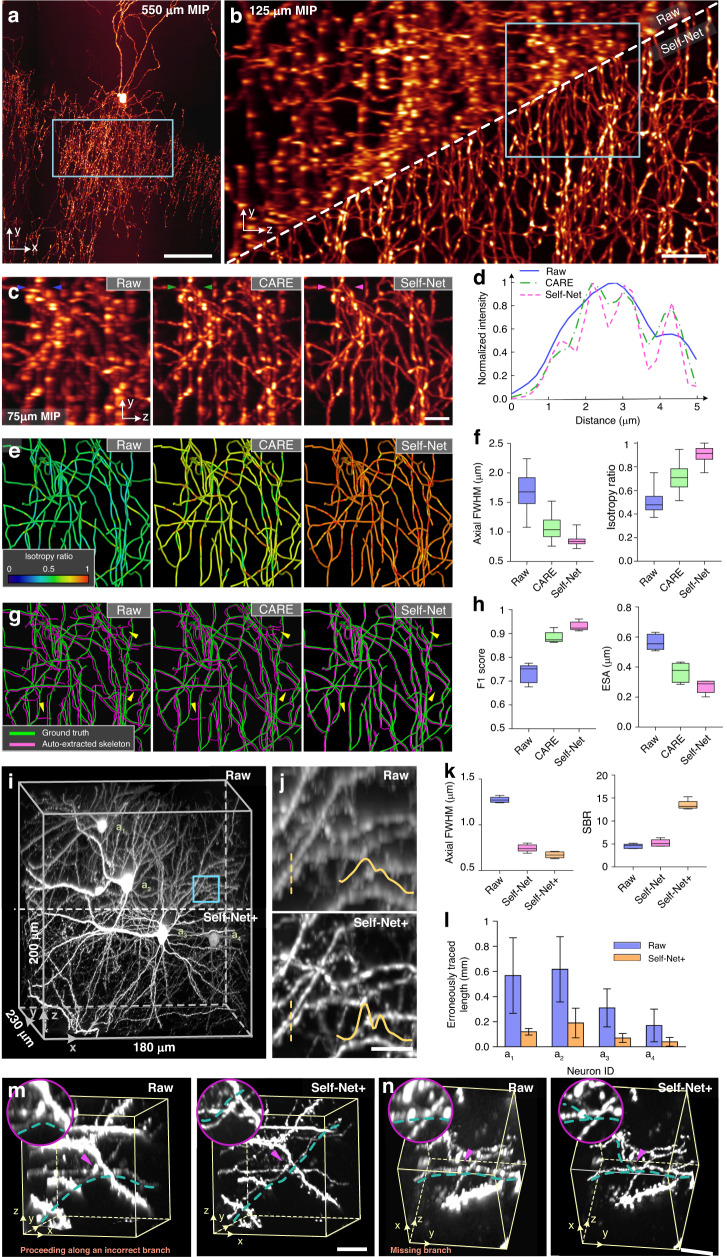
Isotropic restoration via Self-Net facilitates morphology reconstruction of densely-packed neurites. **a** XY MIP of an axo-axonic cell (AAC). Scale bar: 50 μm. **b** YZ MIP of the image volume indicated by the blue box in (**a**). Raw and Self-Net data are shown for comparison. Scale bar: 10 μm. **c** Comparison of the axial MIP images of the raw, CARE, and Self-Net data indicated by the blue box in (**b**). Scale bar: 5 μm. **d** Intensity profiles along the corresponding lines indicated by the colored arrowhead pairs in (**c**). **e** Color-coding isotropy ratios of the images shown in **c**. **f** Axial FWHMs and isotropy ratios of the axon fibers in the image data shown in (**c**). **g** Automated skeletonization of the image data shown in (**c**). The yellow arrowheads indicate typical skeletonization errors when using the raw and CARE data. **h** Automated skeleton extraction accuracy (*n* = 5 subblocks of 300^3^ voxels). **i** Volume rendering of a local dense image block containing four interwoven neurons. Raw and Self-Net+ data were shown for comparison. **j** Local enlarged views indicated by the blue box in **i**. Intensity profiles of the colored dashed line are shown in the bottom right. Scale bar: 5 μm. **k** Average axial FWHMs and signal-to-background ratios (SBRs) for the raw data and Self-Net and Self-Net+ output (*n* = 5 measurements). **l** Erroneously-traced fiber length for each neuron in the raw and Self-Net+ data (*n* = 3 annotators). The gold-standard tracing results were derived from the reconstruction consensus among three neuroanatomical experts, who independently performed tracing through slice-by-slice editing in the HR lateral views of the raw data. **m**, **n** Two types of typical reconstruction errors occur in using the raw data. The dark cyan dashed lines indicate the tracing results of the same annotator using the raw and Self-Net+ data more than 1 week apart. The tracing results using the Self-Net+ data were consistent with the gold-standard tracing results. Top left: enlarged views of the regions indicated by the pink arrowheads. Scale bar: 10 μm. Experiments were repeated with three other dense image stacks, achieving similar results

To test the reconstruction accuracy of Self-Net in a concrete biological context, we applied Self-Net to reconstruct the morphology of densely-packed neurites via the isotropy improvement. In the first example, Self-Net was used to enhance the imaging data of local interneurons. Compared to other cells, interneurons contain extremely dense axon arbors, leading to a highly difficult and painstaking reconstruction process^36^. We demonstrated a typical axo-axonic cell (AAC) located in the medial prefrontal cortex (Fig. 4a). Raw data were acquired with a chemical sectioning fluorescence micro-optical-sectioning tomography (CS-fMOST) system^6^ at a voxel resolution of 0.2 × 0.2 × 1 μm^3^. The fivefold difference between the axial and lateral sampling rates severely hampered the visualization and analysis of these complex data in 3D view. Therefore, the reconstruction of these neurons relied on careful manual tracing in 2D cross-sectional views^15^; however, this slice-by-slice editing process was time-consuming and unintuitive, requiring approximately a week’s time for a skilled annotator to trace a single AAC. To overcome this challenge, we applied Self-Net for isotropic recovery to allow performing neuron tracing in 3D. The fine intermingled axon fibers of the AACs were indistinguishable in the raw axial views, but easily resolved in 3D after Self-Net restoration (Fig. 4b, S8–S10, and Supplementary Video 5).

We also evaluated the CARE method^24^ as a comparison. The CARE approach pioneered isotropic restoration with semisynthetic training data. Since the accurate theoretical model for degrading the lateral image slices into the real axial image slices is hard to obtain in practice, we used the approximate Gaussian degradation model to generate the training data for implementing the CARE method (Methods). We observed that the CARE method mitigated the effects of PSF anisotropy to a certain extent, but compared with Self-Net, the improvement remained modest (Fig. 4c, d). The possible reason is that the approximate Gaussian degradation model is not as accurate as the degradation model directly learned by the network trained with the real imaging data. In addition, benefiting from the direct use of raw data for training, Self-Net avoids the cumbersome process of generating semisynthetic training data in the CARE workflow, resulting in higher application efficiency. We calculated the ratio of lateral to axial FWHM values of the neural fibers as the isotropy ratio (Fig. 4e), and the color-coding results intuitively demonstrated that Self-Net successfully removed the PSF anisotropy of the raw data and outperformed CARE. Self-Net reduced the axial FWHM from 1.68 ± 0.32 to 0.86 ± 0.11 μm, matching the lateral FWHM, and thus improved the isotropy ratio from 0.51 ± 0.10 to 0.91 ± 0.07. In contrast, the CARE network yielded an isotropy ratio of 0.72 ± 0.11, which was substantially inferior to Self-Net (Fig. 4f).

To verify the benefits of the improved resolution isotropy for downstream image analyses, we evaluated the effectiveness of automatic skeleton extraction^37^ (Fig. 4g). Using manually traced skeletons as the GT, we observed that false line segments were common in the raw and CARE data (indicated by the yellow arrowheads in Fig. 4g). Benefiting from the high isotropy ratio and high fidelity, skeleton extraction using Self-Net data yielded high precision reconstruction with center points close to GT. We quantified the accuracy of these auto-extracted skeletons through two standard evaluation metrics, the F1 score (higher scores indicating better accuracy) and the entire structure average^38^ (ESA; lower values indicating better accuracy). The results showed that isotropic restoration via Self-Net substantially improved the accuracy of the automatic neuron extraction and the performance was superior to the CARE approach (Fig. 4h). Manual revision of auto-extracted neuron skeletons is highly required due to the imperfection of full-automation neuron reconstruction algorithms. We, therefore, further semi-manually traced the complete morphology of the individual AACs using the Self-Net data. The improved resolution for better observing and analyzing the dense neurites allows us to substantially reduce the tracing time of single complex AAC to only 12 h, a nearly fourfold improvement over the previous 2D reconstruction time using raw anisotropic data^36^ (Supplementary Table 3).

**Fig. 5:**
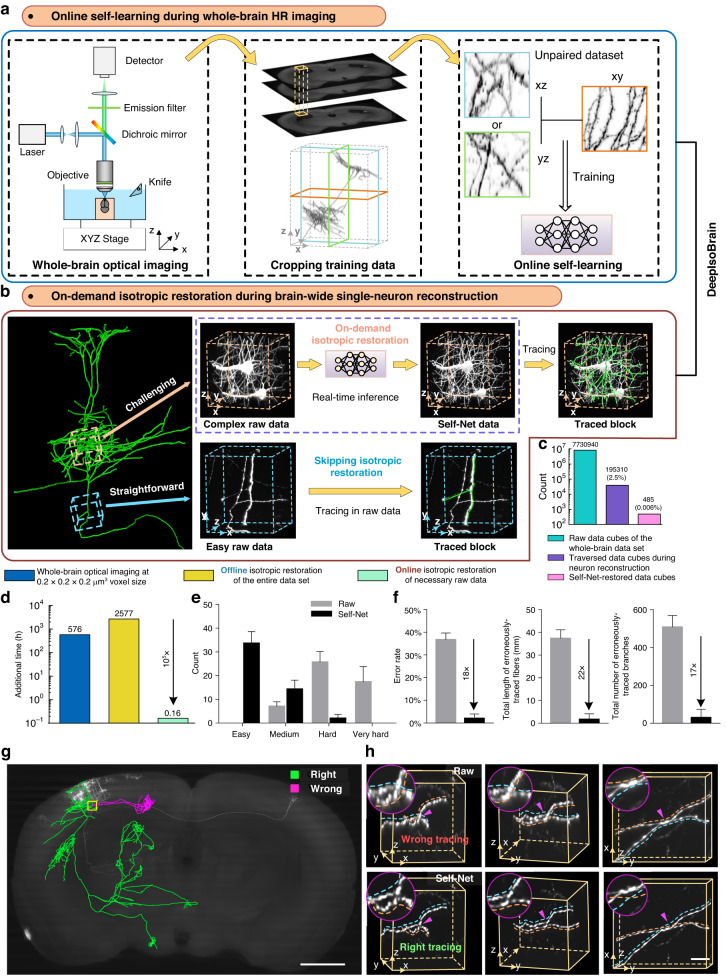
DeepIsoBrain for isotropic whole-brain imaging and accurately reconstructing long-range projection neurons. **a** Online self-learning during whole-brain HR imaging. A data block with 500^3^ voxels containing dense neurites is sufficient for training Self-Net. **b** On-demand isotropic restoration during brain-wide single-neuron reconstruction. **c** The number of raw data cubes (200^3^ voxel size) of the whole-brain dataset, and the traversed and Self-Net-restored raw data cubes while reconstructing all 20 neurons. **d** Additional time cost comparison using different isotropic strategies from the current highest of 0.2 × 0.2 × 1 μm^3^ to 0.2 × 0.2 × 0.2 μm^3^ for whole-brain imaging. **e** Comparing the tracing difficulties in 50 randomly-selected challenging cases using the raw and Self-Net data. **f** The tracing error rate in the challenging regions and the length and branch number of erroneously-traced fibers using the raw and Self-Net data. The tracing GTs for these challenging areas were derived from the reconstruction consensus among three neuroanatomical experts, who independently performed tracing through slice-by-slice editing in the HR lateral views of the raw data. **g** A typical serious reconstruction error of tracing a neuron in the raw data. The accurately and erroneously-traced axon fibers are colored green and pink, respectively. Scale bar: 1 mm. **h** Three orthogonal perspectives of the image block indicated by the yellow box in (**g**) using the raw data and Self-Net-restored data. The dashed lines indicate the tracing results of the same annotator and the colors indicate different branches. The insets show enlarged views of the regions indicated by the magenta arrowheads. The tracing results using Self-Net data were consistent with the gold-standard tracing results. Scale bar: 10 μm

In the second example, we applied Self-Net to restore imaging data of dense neuron clusters, which commonly existed in whole-brain neuron imaging (Fig. 4i). The axial views of the raw data exhibited both low resolution and contrast due to the high-fluorescence background in dense areas. To improve them simultaneously, we employed deconvolution^39^ to further suppress the out-of-focus light in the Self-Net output. To accelerate this two-step process, we trained an end-to-end network (denoted as Self-Net+) using paired raw axial images and deconvolved output images from Self-Net (Fig. S11; Methods). Both Self-Net and Self-Net+ produced output data with an enhanced axial resolution, while the output of Self-Net+ demonstrated a threefold increase in the signal-to-background ratio (SBR) over that of Self-Net (Fig. 5j, k and Supplementary Video 6).

To demonstrate that the substantially improved image quality produced by Self-Net+ can effectively facilitate downstream analyses, we compared the accuracy and efficiency of neurite tracing between the use of raw and Self-Net+ data via the semiautomatic reconstruction module of GTree software^40^. The results revealed that the erroneously-traced fiber length decreased by 4 times (Fig. 4l) and the tracing speed was increased by 53% (Fig. S12) on average by using the Self-Net+ data. We showed two typical reconstruction errors, proceeding along incorrect branches and missing branches, resulting from the poor Z-resolution and low contrast in the raw data (Fig. 4m, n and Supplementary Videos 7, 8). In contrast, Self-Net+ provided high observability in all orientations and allowed the annotators to readily identify the appropriate viewing angle and determine the correct link patterns. In addition, Self-Net+ also benefited the analysis of neuronal substructures such as dendrite spines (Fig. S13). All these results indicated that Self-Net produced reliable isotropic restoration and facilitated common downstream analyses.

### DeepIsoBrain improves morphology reconstruction of brain-wide long-range projection neurons

The success of Self-Net on local imaging data motivated us to address the last-mile problem of data quality in the morphology reconstructions of single neurons due to the resolution anisotropy problem of HR whole-brain imaging. The easy-to-train and high-fidelity characteristics of Self-Net allow us to realize online training of an exclusive network for each whole-brain dataset (Fig. 5a). This strategy not only avoided the additional time for post-training but also overcame the generalization problems of traditional deep learning. We validated this strategy on the HR whole-brain imaging of five mouse brain samples with different fluorescence labels using different imaging systems and showed that each exclusive Self-Net provided nearly isotropic resolution (Supplementary Table 4). The existence of densely tangled neurites is the key factor limiting the accuracy and efficiency of single neuronal reconstruction. The fast inference speed of Self-Net (1.2 s for a 200^3^-voxels data block) enabled us to perform on-demand isotropic resolution recovery of local challenging data in real-time (Fig. 5b), thereby avoiding computationally intensive processing to restore the entire TB-size imaging data. Specifically, to achieve this strategy, we packaged the inference module of Self-Net and integrated it with 3D neuron reconstruction software (e.g., GTree software^40^; Supplementary Video 9), so that as the annotators traverse each data cube along the extension direction of the neurites, they can observe and reconstruct densely-interweaving neurites with isotropic high resolution. We named the combination pipeline of the online self-learning and on-demand isotropic recovery strategies as the DeepIsoBrain method for providing isotropic whole-brain imaging and facilitating single-neuron morphology reconstruction.

To validate the feasibility of DeepIsoBrain, we imaged and traced a whole mouse brain with sparsely labeled pyramidal neurons in the primary somatosensory cortex (S1). The raw data were acquired at the current highest voxel resolution of 0.2 × 0.2 × 1 μm^3^ over 10 days (corresponding to a raw data size of 22 TB). During imaging, we randomly cropped an image stack (500^3^ voxels) containing abundant signals for online training of the Self-Net. After the acquisition, we recruited five annotators to reconstruct all 20 labeled neurons (Fig. S14) and manually recorded areas requiring isotropic restoration. A total of 485 local cubes with 200^3^ voxels (Supplementary Video 10) were applied isotropic resolution enhancement, which only accounted for 0.006 and 0.24% of the whole dataset and traversed data during neuron reconstruction, respectively (Fig. 5c). Obtaining the isotropic HR whole-brain dataset with hardware or computational methods would require an additional 576 and 2577 h, respectively, over standard data acquisition (Fig. 5d). The data size would also reach 110 TB per brain, exponentially increasing the costs and difficulties in subsequent data processing and analysis. In contrast, for DeepIsoBrain, real-time restoration of all local complex areas only required an additional computation time of 10 min (four orders of magnitude lower than before) and did not require saving extra data in the process. Therefore, our approach has successfully addressed the bottleneck problem of data quality in HR whole-brain imaging with minimal effort and cost.

To further evaluate the necessity of isotropic resolution in challenging areas during brain-wide neuron tracing, we compared the tracing difficulties and accuracy between the use of raw and Self-Net data. We randomly selected 50 local areas of densely-interweaving neurites to generate a test dataset of randomly-shuffled raw and Self-Net data. In this blind study, we recruited three skilled annotators to independently trace all test data. More than two levels of tracing difficulty reduction after isotropic recovery (Fig. 5e) indicated the isotropic HR data significantly lowered the complexity of interpreting dense imaging data. Moreover, any local misidentifications may accumulate to escalate the reconstruction errors for long-range projection neurons. We demonstrated that the tracing errors, in this case, were reduced by about 20 times by using Self-Net-restored data (Fig. 5f and Supplementary Table 5). As a typical example, we showed a local decision error leading to the incorrect tracing of more than 20% of the overall length of a projection axon (Fig. 5g and Supplementary Video 11) and 242 subsequent misassigned branches, resulting in unreliable downstream morphological analysis. After isotropic restoration for this local challenging region, the neurite structures were clear in all 3D perspective views (Fig. 5h). The annotators could easily identify the correct proceeding direction of each neurite, thus reducing the occurrence of severe reconstruction errors. These results demonstrated the ability of DeepIsoBrain to assist in efficient and accurate brain-wide single-neuron reconstruction.

## Discussion

In summary, we have presented a general deep learning method, Self-Net, to realize fast and high-fidelity isotropic resolution restoration from organelle to tissue levels for a wide range of microscopy platforms, including wide-field, laser-scanning, and super-resolution microscopy. Moreover, the high effectiveness and applicability of Self-Net enables isotropic HR whole-brain imaging and thus addresses the last-mile problem of data quality in single-neuron morphology reconstruction with an almost negligible cost. Since most biological samples are inherently 3D, our easy realization of isotropic 3D imaging across different microscopy platforms is promising to encourage discoveries of new biological insights.

Our method has overcome the limitations of current neural networks and opens a simple and reliable way to achieve 3D isotropic imaging on different microscope systems. First, obtaining high-quality and precisely-matched training data is difficult or sometimes impossible in practice. We employed the PSF anisotropy and the repeatedly-appearing signal features in arbitrary orientations to develop the self-learning strategy. By employing the natural HR lateral images to serve as rational learning targets, Self-Net does not require prior knowledge of the image formation process and other complex data preparation required by supervised and semi-supervised approaches. Second, for the difficulty of ensuring reliable inference under unsupervised settings, Self-Net incorporates a two-stage process to impose strong supervision on the restoration results that successfully mitigates the hallucination problem owing to the weak constraint in traditional unsupervised methods. This is vital as only high-fidelity reconstruction can be trusted in practical applications. Finally, training and testing of large networks are time-consuming, memory-intensive, and experience-dependent. In contrast, Self-Net is a fast and compact plug-and-play method, and its training process is simple and straightforward without hyperparameter adjustment. All these features make our method more reliable and friendly to use in real-world applications. Distinctly different from other sophisticated hardware solutions, our high-fidelity computational approach can conveniently extend to general laboratories as a routine operation.

As a concrete typical application, the accurate analysis of the complex morphology of single neurons has demonstrated the importance of 3D resolution isotropy for biological research. Several recent works have demonstrated the importance and uniqueness of brain-wide single-neuron morphological reconstruction for revealing projection pathways and defining cell types^36,41–47^. However, the resolution anisotropy of current HR whole-brain imaging still restricts the ability to reconstruct single neuronal morphology accurately and efficiently. The fast, easy-to-train, and high-fidelity features of Self-Net enable us to develop DeepIsoBrain to break the aforementioned bottleneck from the data source in a highly economical manner. Our method can be easily compatible with existing neuronal reconstruction pipelines and offer significant improvements in accuracy and efficiency. This is important for the cell typing study that every systematic work involved thousands of single neuronal reconstructions^41,42^. More importantly, the negligible time and economic cost of our method make it suitable for large-scale, industrialized cell censuses such as BICCN^48^. We anticipate that this method will greatly benefit the construction of single-neuron connectomes and the produced high-quality isotropic data potentially promote the development of automatic algorithms for single-neuron reconstructions.

Considering the ill-posed nature of the inverse problem of restoring the HR images from the LR observations, any network output is a prediction that cannot precisely match the GT image in every detail. Self-Net is no exception, albeit we demonstrated that it can mitigate significant hallucinations in inference. As the tolerance for error is ultimately dependent on the analysis being made, when applying Self-Net to actual imaging data of biological samples, it is recommended to verify the accuracy of the model in specific tasks, such as using the analysis results of human experts in the original data as the GT to verify whether the output results of Self-Net on specific image analysis tasks are consistent with the GT. It should also be noted that all methods which utilize lateral images to improve the corresponding axial images (including Self-Net) rely on a key assumption that the samples appear similar in both lateral and axial views, although most biological structures satisfy this condition. Moreover, the performance of Self-Net deteriorates as the signal-to-noise ratios (SNRs) of the input signals decrease (Fig. S15), suggesting denoising the low SNR data before training Self-Net has the potential to achieve high-quality isotropic recovery.

Overall, the Self-Net deep learning framework is an effective and reliable approach to achieving resolution isotropy of volumetric fluorescence microscopy. We anticipate that Self-Net will be useful in a variety of fields to improve not only existing 3D imaging datasets but also new data acquisitions, and the general idea behind it may be feasible for other image restoration tasks besides isotropic recoveries, such as unsupervised denoising and background suppression.

## Materials and methods

### Generation of the synthetic and semisynthetic datasets

#### Simulation of the bead volumes

In Fig. 1b, the GT image volumes containing beads at random locations and with random radius and intensity values were generated through customized Python scripts. The radii of the beads ranged from 2 to 4 pixels, and the gray values ranged from 150 to 255. The volume for training isotropic recovery models contained 800 beads and had a spatial size of 512 × 512 × 512 pixels. We defined the pixel size as 200 nm. The widefield PSF with 1.0-NA and water immersion was generated using the ImageJ plugin PSF generator^49^. The anisotropic image volumes were obtained by convolving the GT image volumes with the wide-field PSF.

#### Simulation of the tubular volumes

In Fig. 1c, the 1024 × 1024 × 1024 voxels-size synthetic volume containing 5000 tubular objects was generated using the code released in ref. ^26^. The GT data were generated by convolving the synthetic tubular volume with an isotropic Gaussian kernel that had a standard deviation of 1. The anisotropic image volume was produced by convolving the same tubular volume with an anisotropic Gaussian kernel that laterally and axially had a standard deviation of 1 and 4, respectively. This anisotropic image stack was further downsampled along the axial direction with a scaling factor of 4 for mimicking the subsampling process in real-world volumetric acquisitions.

#### Generation of semisynthetic training data

In Fig. 1g, several image blocks containing dense neurites from a randomly-selected whole-brain optical imaging dataset were cropped. Lateral slices of the data blocks were used as HR targets. The pixel-aligned anisotropic blurred images were generated by first convolving these HR images with a 2D Gaussian kernel that only blurs in the vertical direction with a standard deviation of 2. Then the anisotropic blurred images were downsampled along the vertical direction at a scale factor of 4 (Fig. S3).

### Sample preparation and data acquisition

#### 3D imaging of diverse biological samples

One mTmG mouse was used in Fig. 2a, b. About 200-μm tissue slices of the liver and 300-μm tissue slices of the kidney were optically cleared by CUBIC^50^. The liver slices were imaged using a custom-built time-delay integration-based line-scanning microscope^6^ that has the characteristics of wide-field imaging and enhanced signal-to-noise ratio (Fig. 2a). A 20× /1.0-NA water-immersion objective (XLUMPLFLN 20XW, Olympus) was used for acquisition and the voxel size was 0.32 × 0.32 × 1 μm^3^. The kidney slices were imaged using a Nikon Ni-E A1 multiphoton laser-scanning microscope with a 60× /1.2-NA water-immersion objective (Nikon) using 920-nm two-photon excitation and a voxel size of 0.21 × 0.21 × 1 μm^3^ (Fig. 2b). The whole-brain vasculature datasets used in Fig. 2c were provided by Todorov et al.^28^, where the 3DISCO^51^ cleared brains were acquired with LaVision light-sheet microscopes at a 1.63 × 1.63 × 3 μm^3^ voxel resolution. One Thy1-GFP M-line mouse was used in Fig. 2d. The mouse brain was sectioned into 300 μm tissue slices. The sections were optically cleared by CUBIC and then imaged using the Nikon Ni-E A1 microscope working in the confocal mode. The imaging setup was the same as the two-photon imaging of the kidney.

#### STED imaging

The 40-nm fluorescent beads (abberior NANOPARTICLE SET) were imaged on a STED microscope (STED Abberior Facility Line) using a 60× /1.4-NA oil-immersion objective. The STED laser power was set at 50%, as we observed obvious photobleaching when performing 3D imaging at 100% laser power. The Z-STED data was acquired using 100% depletion power for Z. The same FOV was then imaged using 30% depletion power for Z and 70% depletion power for XY (Partial Z-STED). The scanning step size for acquiring the Z-STED data were 30 nm in all dimensions and for Partial Z-STED was 15 nm laterally and 50 nm axially.

#### Whole-brain imaging

Two Nkx2.1-CreER-Rosa26-loxp-stop-loxp-flpo (Nkx2.1-CreER crossed with Rosa26-loxp-stop-loxp-flpo) mice with sparsely virus-labeled AACs were used to demonstrate the performance of Self-Net on local interneurons (Fig. 4a–h and S8–S10). Two C57BL/6 J mice injected with 100 nL of AAV-YFP in S1 were used to demonstrate that Self-Net promoted morphology reconstruction of dense neuron clusters (Fig. 4i–n) and long-range projection neurons (Fig. 5). One C57BL/6 J mouse injected with 100 nl of AAV2-hSyn-FLEX-pHOran4 in the S1 and M1 cortex was used for dendrite spines detection study (Fig. S13). All mice were obtained from Jackson Laboratory and were raised to adulthood (>8 weeks) before the imaging experiments. The sample preparation procedures have been described previously^52^. All animal experiments followed procedures approved by the Institutional Animal Ethics Committee of the Huazhong University of Science and Technology.

The datasets used in Figs. 4–5 and S8–S10 were acquired with two fMOST systems^6^ at a 0.2 × 0.2 × 1 μm^3^ voxel resolution. Whole-brain imaging in Fig. S13 was performed on a fMOST system at a 0.235 × 0.235 × 1 μm^3^ voxel resolution.

### Self-Net for isotropic restoration

Different from self-supervised learning^17,24^, Self-Net employs the self-learning strategy^53,54^ for learning isotropic restoration only using the raw 3D anisotropic data itself. It does not require the acquisition of isotropic target data or handcrafted models to generate semisynthetic training data. The training flowchart for Self-Net is shown in Fig. S16. Consider an anisotropic image volume: *X* denotes the set of HR lateral images, and *Z* denotes the set of blurred axial images. Due to the low sampling rate along the axial direction, all images in *Z* are interpolated to have the same pixel size as that of the images in *X*. *Y* denotes the set of downsampled lateral images, which are generated by first downsampling the images in *X* along the vertical direction at a certain scale (the axial step divided by the pixel size in the lateral plane) and then interpolating these images to the original size. Image *x*_*i*_ in *X* is paired with image *y*_*i*_ in *Y* and unpaired with image *z*_*i*_ in *Z*.

Self-Net is a two-stage unsupervised framework for high-fidelity isotropic restoration in volumetric microscopy. The first stage performs degradation modeling, and the second stage performs isotropic recovery. At the degradation modeling stage, similar to the CycleGAN framework, two generators (*G*_*A*_ and *G*_*B*_) and two discriminators (*D*_*A*_ and *D*_*B*_) are trained to learn the mapping between the unpaired datasets *Y* and *Z*. For the *Y* to *Z* mapping, *G*_*A*_ aims to degrade the downsampled lateral image *y*_*i*_ to simulate the blurred axial image *z*_*i*_, and *D*_*A*_ attempts to distinguish the synthetic blurred axial image *G*_*A*_(*y*_*i*_) from the real axial image *z*_*i*_. For the opposite mapping (*Z* to *Y*), *G*_*B*_ takes the axial image *z*_*i*_ as input to generate images that resemble the downsampled lateral images *y*_*i*_, and *D*_*B*_ attempts to distinguish the generated image *G*_*B*_(*z*_*i*_) from the real image *y*_*i*_. The objective function of this adversarial training framework *L*_GAN_ can be defined as:

1 $$\left\{\begin{array}{l}{L}_{GAN}{={\rm{L}}}_{G}+{L}_{D}\\ {L}_{G}=\frac{1}{N}\mathop{\sum }\limits_{i=1}^{N}{\Vert {D}_{A}[{G}_{A}({y}_{i})]-1\Vert }_{2}+\frac{1}{N}\mathop{\sum }\limits_{i=1}^{N}{\Vert {D}_{B}[{G}_{B}({z}_{i})]-1\Vert }_{2}\\ {L}_{D}=\frac{1}{N}\mathop{\sum }\limits_{i=1}^{N}{\Vert {D}_{A}[{G}_{A}({y}_{i})]-0\Vert }_{2}+\frac{1}{N}\mathop{\sum }\limits_{i=1}^{N}{\Vert {D}_{A}({z}_{i})-1\Vert }_{2}\\ +\frac{1}{N}\mathop{\sum }\limits_{i=1}^{N}{\Vert {D}_{B}[{G}_{B}({z}_{i})]-0\Vert }_{2}+\frac{1}{N}\mathop{\sum }\limits_{i=1}^{N}{\Vert {D}_{B}({y}_{i})-1\Vert }_{2}\end{array}\right.$$

where *L*_G_ is the generator loss, *L*_D_ is the discriminator loss, and *N* is the batch size. Note that the least-square loss is employed instead of the negative log-likelihood loss to achieve stable training^55^. To prevent mode collapse, the generator loss is updated twice and then the discriminator loss is updated once in each iteration. Besides the adversarial training loss, the cycle consistency loss *L*_cycle_ is introduced to constrain the space of possible mappings and can be defined as:

2 $${L}_{cycle}=\frac{1}{N}\mathop{\sum }\limits_{i=1}^{N}{\Vert {G}_{B}[{G}_{A}({y}_{i})]-{y}_{i}\Vert }_{1}+\frac{1}{N}\mathop{\sum }\limits_{i=1}^{N}{\Vert {G}_{A}[{G}_{B}({z}_{i})]-{z}_{i}\Vert }_{1}$$

Instead of directly using the L1 loss function, the robust Charbonnier loss^56^ is employed for better-handling outliers.

At the isotropic recovery stage, the weights of *G*_*A*_ are fixed. The synthetic blurred axial image *G*_*A*_(*y*_*i*_) along with the pixel-aligned HR lateral image *x*_*i*_ are used to train DeblurNet, here denoted as *H*. The training loss *L*_deblur_ is the weighted sum of the L1 Charbonnier and SSIM losses:

3 $${L}_{{\rm{de}}blur}=\frac{1}{N}\mathop{\sum }\limits_{i=1}^{N}\Big\{{\Vert H[{G}_{A}({y}_{i})]-{x}_{i}\Vert }_{1}+\sigma (1-SSIM(H[{G}_{A}({y}_{i})],{x}_{i}))\Big\}$$

where *σ* is the weighted term and is chosen as 0.1.

To enhance the performance of the degradation modeling stage, the results obtained at the isotropic recovery stage are introduced via feedback to help guide network *G*_*A*_ to generate more realistic degraded images. The feedback loss *L*_feedback_ shares the same form as that of the deblurring loss *L*_deblur_. The only difference is that the weights of *H* instead of *G*_*A*_ are fixed. Taken together, the full objective at the degradation modeling stage can be written as:

4 $${L}_{{\rm{degrade}}}{={\rm{L}}}_{{\rm{cy}}cle}+\lambda {L}_{GAN}+\rho {L}_{feedback}\,$$

where *λ* and *ρ* are weighted terms. Similar to the settings in the original CycleGAN^25^, the weighted term *λ* for balancing the cycle consistency loss and GAN loss is set to 0.1. An appropriate weight for the feedback loss is important to obtain a satisfactory isotropic recovery performance. After careful comparison, *ρ* is set to 0.1 (Fig. S17). The degradation modeling loss *L*_*degrade*_ and deblurring loss *L*_deblur_ are alternatively optimized for stable training and fast convergence.

### Network architecture

The architectures of all networks in Self-Net are illustrated in Fig. S18. DeblurNet, DeconvNet, and the generators (*G*_*A*_ and *G*_*B*_) share the same architecture and are constructed with residual blocks^57^. Different from the image transform net employed in the CycleGAN, we removed the downsampling and upsampling modules as they were designed for the style transfer task. We also employed fewer residual blocks to speed up network inference. Specifically, The initial part of the network contains three convolutional layers, each of which is followed by a leaky rectified linear unit (LeakyReLU)^58^ with a negative slope of 0.2, formulated as:

5 $$L{\rm{ea}}kyRELU(x)=\left\{\begin{array}{l}x,x \,>\, 0\\ 0.2x,x\,\le\, 0\end{array}\right.$$

The middle part of the network comprises six residual blocks, each of which contains two convolutional layers with LeakyReLU activation functions. The operation for each residual block can be denoted as:

6 $$y=x+LeakyRELU[Con{v}_{2}(LeakyRELU[Con{v}_{1}(x)])]$$

where *x* is the input tensor, *y* is the output tensor, and Conv(.) denotes the convolution operation. Symmetrical to the beginning part, the tail of the network also contains three convolutional layers, where the outputs of the first two convolutional layers are activated via the LeakyReLU function. Except for the first and last convolutional layers, which use 7 × 7 kernels, the remaining convolutional layers all employ 3 × 3 kernels. For all convolutional layers, a 1 × 1 stride and zero-padding are applied to maintain the initial image size. The feature size is set to 64 across all convolutional layers except the last layer, in which the feature size is set to 1.

The discriminators (*D*_*A*_ and *D*_*B*_) are based on the PatchGAN framework^59^. Their networks contain four convolutional layers with a 4 × 4 kernel size; the first two layers use a 2 × 2 stride, while the last two layers use a 1 × 1 stride. Each pixel in the output image reflects the probability that a 34 × 34 patch in the corresponding input image is real or fake. The first convolutional layer is followed by a LeakyReLU layer, and each of the next two convolutional layers is followed by an instance normalization layer^60^ and LeakyReLU layer. The feature size is set to 64, 128, 256, and 1 for the four convolutional layers in order.

### Training and testing of Self-Net

Self-Net is trained on image patches with a spatial size of 64 × 64 pixels. All training data are normalized to the 0–1 range following the criterion described in ref. ^24^. Image augmentation, including random rotation and flipping, is employed during training.

The network parameters are optimized via the Adam optimizer^61^ with β_1_ = 0.5 and β_2_ = 0.999. The learning rate for all networks is 1e-4 and decays by half after every 20 epochs. Self-Net training requires approximately 1 h on a single Nvidia GeForce RTX 3090 card (24 GB of memory) for 40 epochs (equivalent to 24,000 iterations). The mini-batch size is set to 8. In the testing phase, only DeblurNet is activated, and the inference time on an image volume with a spatial size of 200 × 200 × 200 pixels is 1.2 s. In all the data presented, the training of the corresponding Self-Net models was under the same hyperparameter settings as described above.

It is worth noticing that for new sample types or new imaging conditions, it is required to use a single 3D stack from the imaging data for training a new Self-Net model to achieve optimal results. And for other 3D stacks under the same conditions with the training data, it is unnecessary to train additional Self-Net models.

### Training and testing of Self-Net+

The Self-Net+ network is proposed to reconstruct high SBR 3D image stacks with isotropic resolution in the presence of strong out-of-focus background. The core idea of this method is to incorporate the deconvolution algorithms to further improve the SBR of the isotropic image stack output by Self-Net. Because as a widely-recognized optical-sectioning method, deconvolution can be used to remove the out-of-focus light by partially reversing the image formation process with knowing PSF and noise prior^39,62^. Since Poisson noise is the dominant noise source in fluorescence microscopy, we employed the RL algorithm for image deconvolution because it assumes that the image noise follows a Poisson distribution. We implemented the RL algorithm using the deconvlucy function in MATLAB (Mathworks). In the experiments of Figs. 4,5, we cropped an isolated diffraction-limited fluorescent dot (50 × 50 pixels size) from the XZ slice of the Self-Net output to serve as the experimental PSF (Fig. S19). After centering, background subtracting, and normalizing, the PSF kernel can be used for RL deconvolution. The number of iterations was set as 5.

The procedure for training Self-Net+ is as follows: (1) Self-Net is trained using the raw image stack; (2) the trained Self-Net is applied to enhance the axial images in the raw image stack; (3) the output of the trained Self-Net is deconvolved with the RL algorithm; and (4) DeconvNet is end-to-end trained with the paired raw axial images and deconvolved Self-Net output images. After training, DeconvNet is loaded to enhance all the axial images in the raw anisotropic volume (Fig. S11).

### Implementation details for OT-CycleGAN and CARE

#### OT-CycleGAN

The OT-CycleGAN framework has been described in detail in ref. ^26^. We replicated this model through the authors’ released code (Zenodo 10.5281/zenodo.6371391). The hyperparameter settings for training and testing on data of fluorescent beads, simulated tubular objects, brain vasculature, and neurons were consistent with the settings in ref. ^26^ on similar corresponding data. As recommended in ref. ^26^, we employed histogram-matching as post-processing to ensure a consistent contrast between the input and network output volumes.

#### CARE

The CARE method for isotropic recovery^24^ incorporates two steps: (1) applying a degradation model (blurring and downsampling) to modify the HR lateral images to resemble LR axial images. (2) using these semi-synthetically generated pairs to learn to reverse the lateral-to-axial degradation. In the experiments in Fig. 4c–h, we applied a Gaussian kernel (sigma = 3 pixels) that only blurs in the *y* dimension to the lateral slices of the AAC imaging data. And the blurred images were then downsampled by a factor of 5 to resemble the LR axial images. The generated anisotropic blurred images, along with the high-resolution lateral view ground truth, were used to train the DeblurNet for learning to reverse this anisotropic degradation.

### Image quality evaluation

Two widely used performance metrics, i.e., RMSE and SSIM^63^, were employed to quantify the distance between the network output *M* and ground truth *N*. In detail, RMSE is defined as:

7 $$RMSE(M,N)=\sqrt{\frac{1}{W\times H}\mathop{\sum }\limits_{i=1}^{W}\mathop{\sum }\limits_{j=1}^{H}{({M}_{i,j}-{N}_{i,j})}^{2}}$$

where *W* and *H* are the image width and height, respectively; a lower RMSE value indicates that the output image is close to the GT image. SSIM can be defined as:

8 $$SSIM(M,N)=\frac{(2{\mu }_{M}{\mu }_{N}+{C}_{1})(2{\sigma }_{MN}+{C}_{2})}{({{\mu }_{M}}^{2}+{{\mu }_{N}}^{2}+{C}_{1})({{\sigma }_{M}}^{2}+{{\sigma }_{N}}^{2}+{C}_{2})}$$

where *µ*_*M*_ and *µ*_*N*_ are the mean values of the two images *M* and *N*, respectively, *σ*_*M*_ and *σ*_*N*_ are the standard deviations of *M* and *N*, respectively, and *σ*_*MN*_ is the covariance between the two images. Two constants *C*_*1*_ and *C*_*2*_ are used to avoid denominator values close to zero. The output value of SSIM varies between 0 and 1, and a value closer to 1 suggests less distortion.

The signal-to-background ratio (SBR) was calculated as SBR = *s*/*b*, where *s* is the peak value of the signal, and *b* is the mean value of the surrounding background.

### Neuron reconstruction and visualization

#### Automatic neuron skeleton extraction

Automatic extraction of the neuron skeleton was implemented via the constrained principal curve algorithm, which is thoroughly described in ref. ^37^. Briefly, for a given image stack, seed points are first selected based on criteria associated with the signal intensity and local background. Next, the initial tracing direction of each seed point is determined via principal component analysis (PCA). Neurite tracing starts from the selected seed points, and the direction of the current traced points is determined according to the previously traced points. To obtain traced points close to the neurite centerline, the mean shift algorithm is applied. Finally, the rayburst sampling algorithm^64^ is employed to decide whether to stop tracing.

#### Isotropy ratio

In Fig. S6, we estimated the isotropy ratio by randomly selecting tubular structures in the imaging data, and then measuring the FWHM values of the same structure in the lateral and axial planes. In Fig. 4e, f, for a given neuronal image stack, we first manually skeletonized neurite fibers. Skeleton points were resampled to obtain equal spacing. We then extracted a series of data cubes of 23 × 23 × 23 voxels size centered on each point in the traced skeleton. The forward direction of each skeleton point was determined by its four nearest neighboring points^65^, and the normal direction through this point was the direction orthogonal to the forward direction. We projected the normal line onto the lateral and axial MIPs of the cube and obtained intensity profiles along these lines. The isotropy ratio at each skeleton point was defined as the ratio of the FWHM value derived from the lateral line-scan profile to the FWHM value derived from the axial line-scan profile. Due to interference from closely spaced neurites, certain points yielded a calculated isotropy ratio that greatly deviated from the actual value. Thus, we first manually measured several sets of isotropy values for the data block and determined the average as a temporary standard. All automatically calculated isotropy ratios deviating by more than one-half of this standard were discarded.

#### Semiautomatic neuron tracing

Since fully automatic neuron reconstruction methods are yet to be reasonably reliable and stable, current recognized methods for single-neuron tracing require manual supervision. Therefore, we employed the semiautomatic mode in GTree software^40^ to reconstruct the morphology of labeled neurons in local image stacks or whole-brain datasets (Figs. 4, 5). Before tracing, TIFF image data were transformed into the native TDat format^66^ to manage the data input-output. The reconstruction process started from the cell body and was performed by traversing and loading all subblocks containing associated neurites. In each subblock, automatic tracing based on the constrained principal curve was performed first, followed by a manual assessment to determine whether to continue automatic tracing or to revise any reconstruction errors. We recorded the operation time during the tracing using customized Python scripts. The reconstruction results were saved as SWC files comprising a series of skeleton points. The gold-standard tracing results were derived from the reconstruction consensus among three neuroanatomical experts, who independently performed tracing through slice-by-slice editing in the HR lateral views of the raw data.

#### Performance metrics

Two commonly used metrics, namely, the F1 score and ESA, were employed to quantify the difference between the tracing results and GT data. Denote R_1_ as the GT and R_2_ as the reconstruction generated by automatic or semiautomatic methods, respectively. Before calculation, the nodes in R_1_ and R_2_ were first resampled to obtain equal spacing. For each node in R_2_, we searched for the node in R_1_ with the smallest distance from it, and this distance was denoted as the reciprocal minimal spatial distance. If the reciprocal minimal spatial distance was lower than a predefined threshold (typically set to 1–3 μm according to the maximum diameter of the neurites), the node in R_2_ was regarded as a true positive (TP) node. The recall and precision rates were defined as the number of TP nodes divided by the number of nodes in R_1_ and R_2_, respectively. Then, the F1 score can be formulated as:

9 $$F1=\frac{2PR}{P+R}$$

where P and R denote the precision and recall rates, respectively. The ESA value was derived by averaging all reciprocal minimal spatial distances. Thus, the lower the ESA value is, the closer the two reconstruction results are.

#### Criteria for tracing difficulty evaluation

To evaluate the tracing difficulty in locally challenging areas, four difficulty levels were defined according to the annotators’ decision times: very hard for a decision time of more than 3 min, hard for a decision time ranging from 2 to 3 min, medium for a decision time ranging from 1 to 2 min and easy for a decision time less than 1 min.

#### Automatic detection of dendrite spines

Dendrite spines were automatically detected using the filament module of Imaris software (V.9.0, Bitplane). To generate the GT, three neuroanatomical experts were recruited to manually identify spines independently. Their consensus was regarded as the GT.

#### Visualization

The 3D volumes were visualized in Amira (v.6.1.1, FEI) and Imaris software to generate figures and movies.

### Supplementary information

Supplementary information

Supplementary video 1

Supplementary Video 2

Supplementary Video 3

Supplementary video 4

Supplementary Video 5

Supplementary Video 6

Supplementary video 7

Supplementary video 8

Supplementary video 9

Supplementary video 10

Supplementary video 11

## Data Availability

The data for isotropic restoration experiments of simulated fluorescent beads, simulated tubular volume, mouse liver, mouse kidney, mouse brain neurons, fluorescent nanobeads, AAC neurons, and dense neuron clusters can be found at 10.5281/zenodo.7882519. The data of light-sheet imaging of mouse brain vessels can be found in ref. ^28^. The data of iSiM imaging of fixed U2OS cells can be found in ref. ^21^. The code for training and testing of Self-Net can be found at 10.5281/zenodo.7882519.
