# Supplementary material for: Deep self-learning enables fast, high-fidelity isotropic resolution restoration for volumetric fluorescence microscopy
Source: Light Sci Appl. 2023 Aug 28;12:204. doi: 10.1038/s41377-023-01230-2 (PMC10462670; doi:10.1038/s41377-023-01230-2)
Supplement: Supplementary file 1 — Supplementary information [file 41377_2023_1230_MOESM1_ESM.docx]

**Supplementary Information**

**Deep self-learning enables fast, high-fidelity isotropic resolution restoration for volumetric fluorescence microscopy**

Kefu Ning^1,2,3^, Bolin Lu^1,2,3^, Xiaojun Wang^1,2,4^, Xiaoyu Zhang^1,2^, Shuo Nie^1,2^, Tao Jiang^3^, Anan Li^1,2,3^, Guoqing Fan^1,2^, Xiaofeng Wang^3^, Qingming Luo^1,2,3,4^, Hui Gong^1,2,3*^, Jing Yuan^1,2,3*^

^1^Britton Chance Center for Biomedical Photonics, Wuhan National Laboratory for Optoelectronics, Huazhong University of Science and Technology, Wuhan, China.

^2^MoE Key Laboratory for Biomedical Photonics, School of Engineering Sciences, Huazhong University of Science and Technology, Wuhan, China.

^3^HUST-Suzhou Institute for Brainsmatics, Suzhou, China.

^4^School of Biomedical Engineering, Hainan University, Haikou, China.

These authors contributed equally: Kefu Ning, Bolin Lu.

*Correspondence to: huigong@hust.edu.cn; yuanj@hust.edu.cn.

**Supplementary Figures**


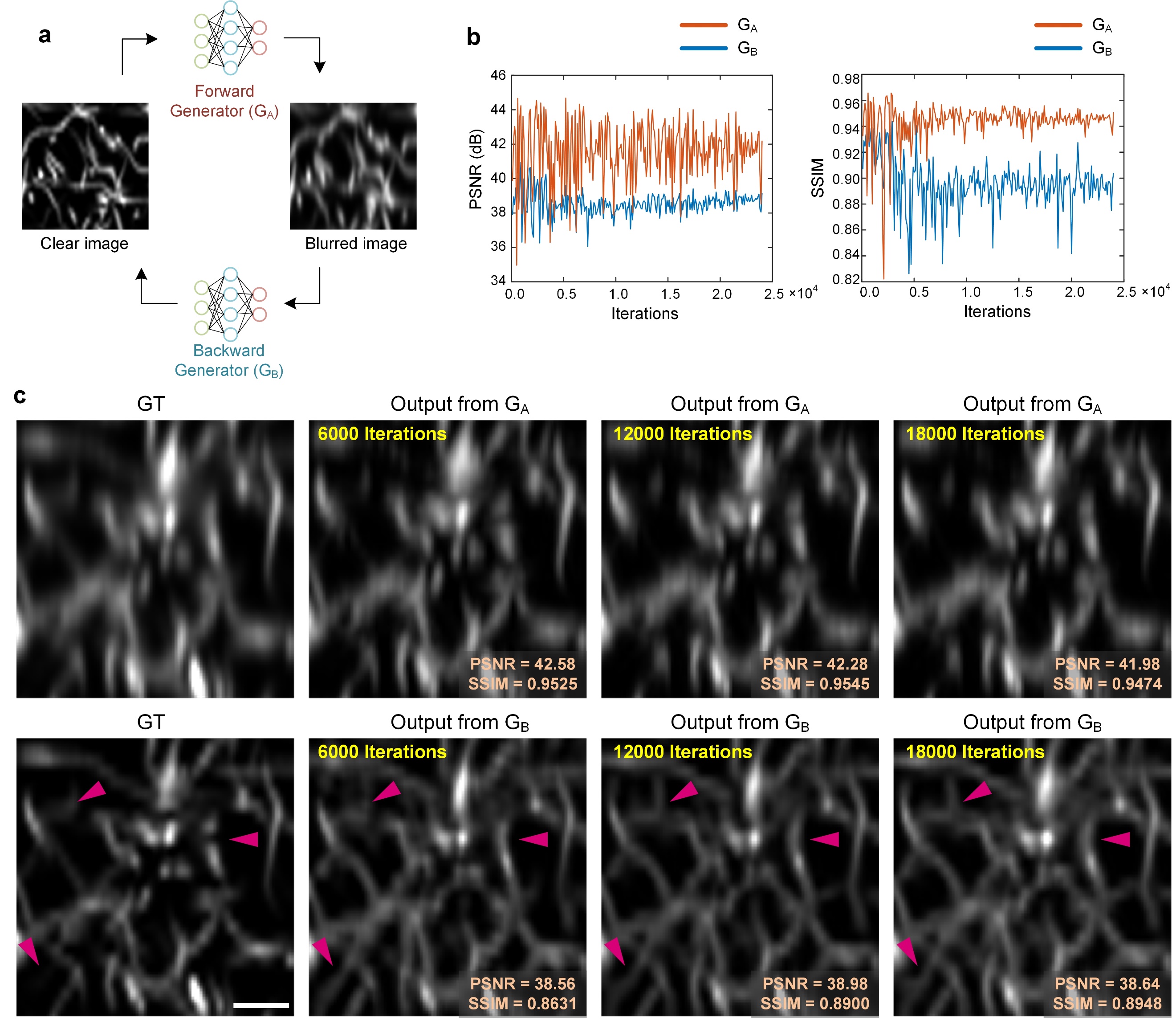


**Fig. S1 Demonstration of the phenomenon that in unsupervised learning, networks learn image degradation better than learn image deblurring. a**, Unsupervised training with CycleGAN to learn the mapping between clear image domain and blurred image domain. Forward generator G_A_ aims to degrade the clear images to generate blurred images. Backward generator G_B_ aims to deblur the blurred images to generate clear images. **b**, The PSNR and SSIM image quality metrics of the intermediate outputs from the G_A_ and G_B_ during unsupervised training. The results show that G_A_ learns significantly better than G_B_, revealing the effect that in unsupervised training, networks learn image degradation better than image deblurring. This result supports our choice to employ unsupervised training to learn the image transformation from the clear image domain to the blurred image domain. **c**, Demonstration of typical intermediate outputs from the G_A_ and G_B_ during unsupervised training. The colored arrowheads highlight the obvious hallucinations in the outputs from G_B_, indicating the potential risks of directly reconstructing clear images using the unsupervised CycleGAN framework. Scale bar, 20 pixels.


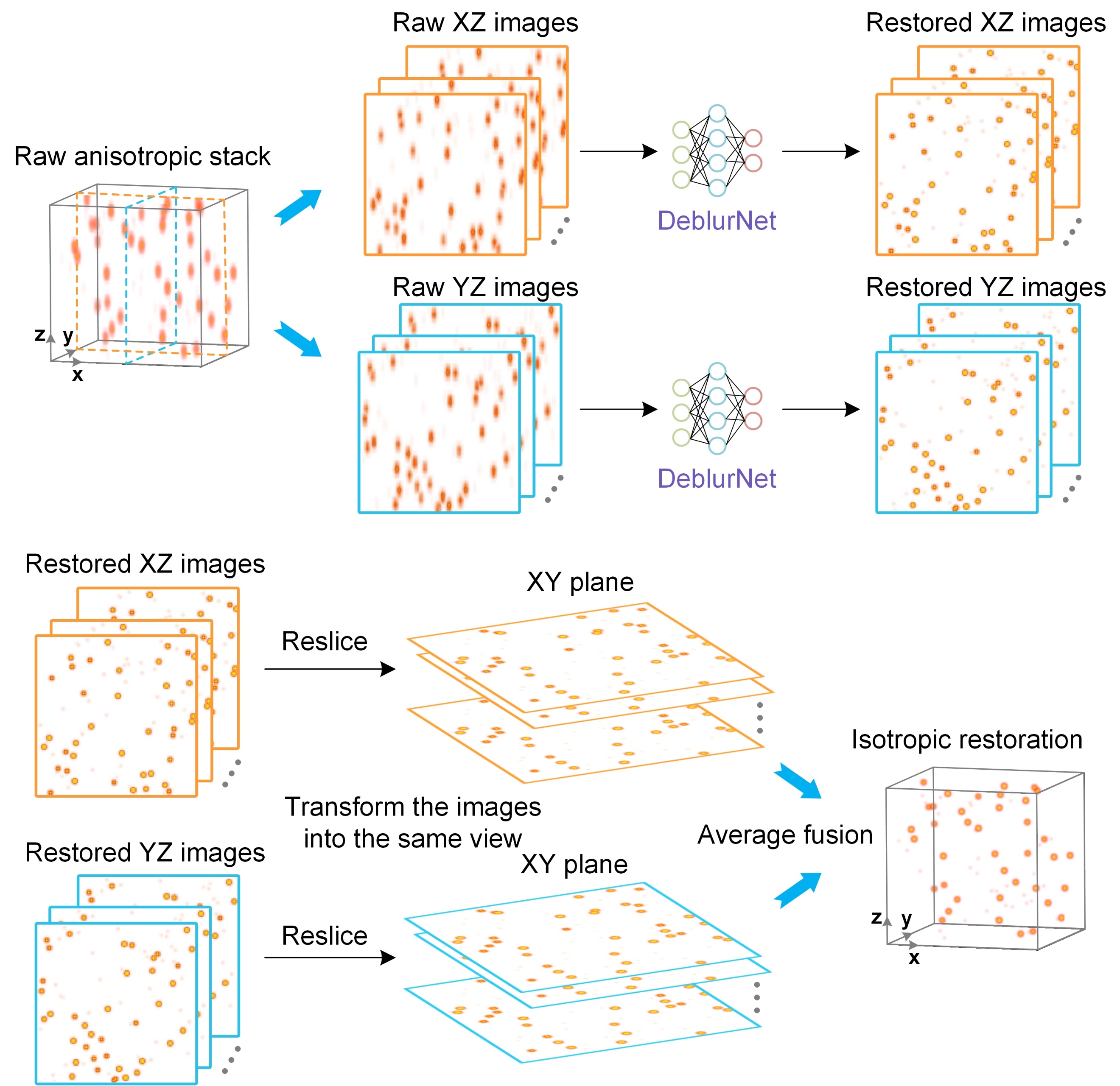


**Fig. S2 Self-Net reconstruction pipeline.** Raw axial slices obtained from two orthogonal directions (XZ and YZ) are first enhanced by DeblurNet and then averaged to generate the final output.


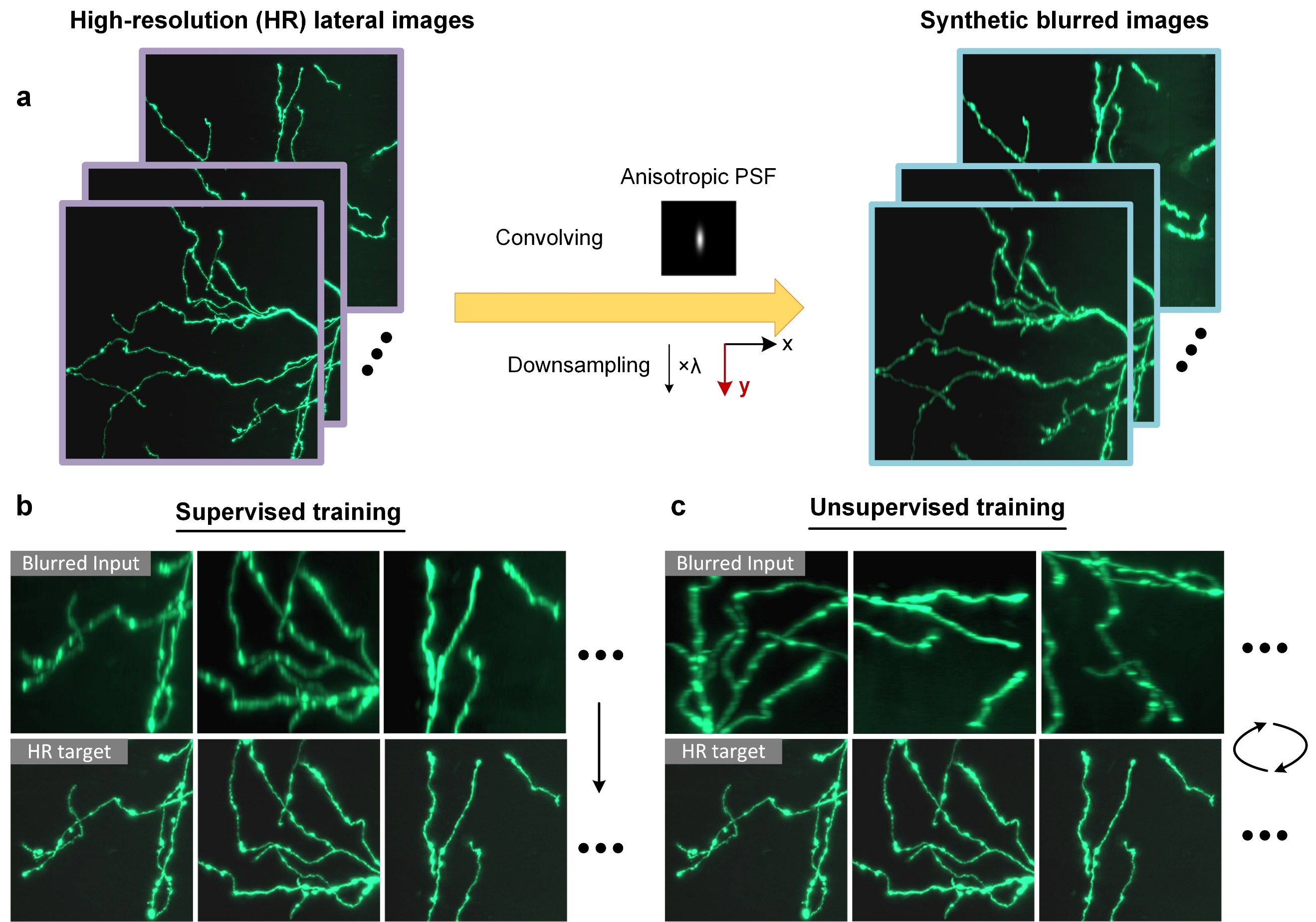


**Fig. S3 Generation of semisynthetic datasets for supervised and unsupervised training. a**, Application of a predefined degradation model to the high-resolution (HR) lateral images to generate synthetic blurred images. The degradation model includes 1) blurring of the HR images by an anisotropic Gaussian PSF and 2) downsampling of the blurred images along the y-direction at scale λ and then interpolating to the original size. **b**-**c,** Network training in supervised (b) and unsupervised (c) manners.


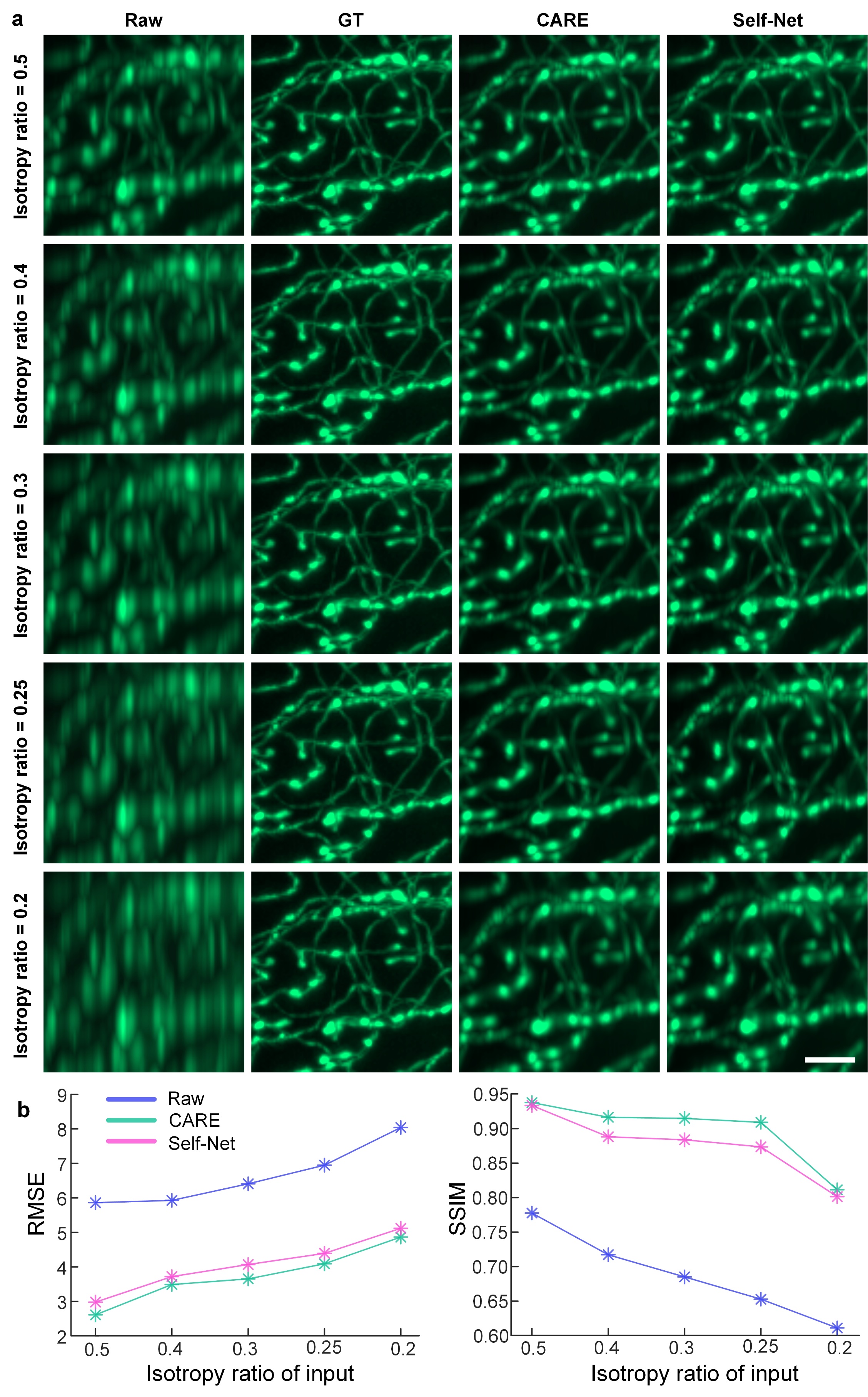


**Fig. S4 Network performance evaluation under different extents of resolution anisotropy. a**, Rows (from top to bottom): results obtained under different extents of resolution anisotropy of the input. The HR image data is the same as used in Fig. 1g. The isotropy ratio is defined as the ratio of lateral to axial resolution. The level of isotropy ratio was controlled by the combination of different degrees of Gaussian blur and down-sampling rates. Columns (from left to right): Demonstration of a typical area of the anisotropic input, ground truth (GT), CARE output (supervised training), and Self-Net output (unsupervised training). Scale bar, 5 μm. **b**, Average RMSE and SSIM quality metrics of different methods as a function of isotropy ratio (n=5). The results demonstrated that the isotropic recovery performance of both networks suffered from noticeable deterioration as the input isotropy ratio is below 0.25.


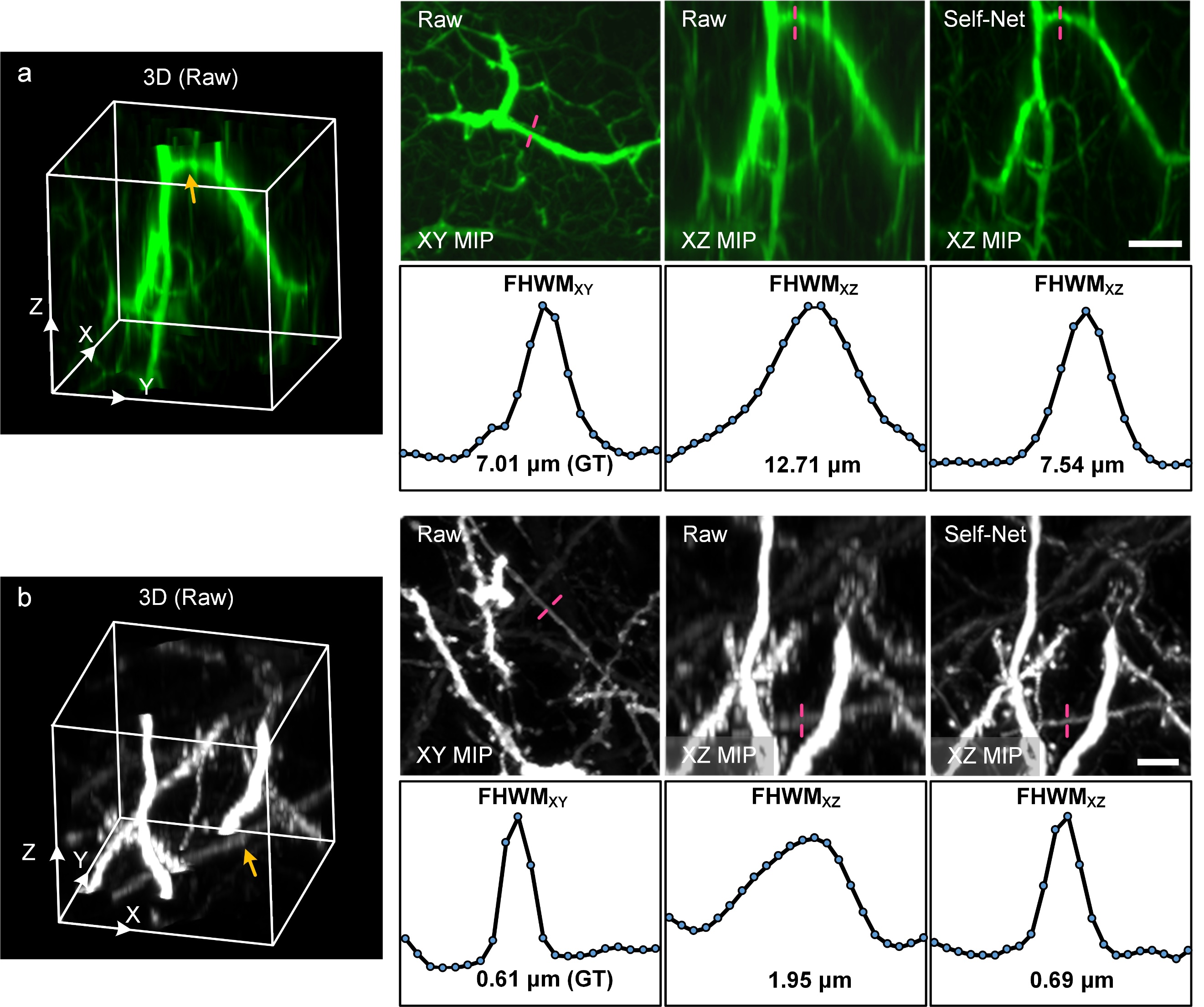


**Fig. S5 Validating the accuracy of resolution enhancement by Self-Net using the estimation in the corresponding lateral view as GT.** a-b, Light-sheet and confocal imaging of cleared mouse brain vasculature (a) and neurons (b), respectively. The 3D volume renderings of the raw image stacks are shown on the left. The orange arrows indicate the structures for FWHM analysis. First row (from left to right): The XY and XZ MIP of the raw image stack, and the XZ MIP of the Self-Net restoration. Second row: The corresponding intensity profiles along the red dashed lines in the images shown in the first row. The FHWMs of the same vessel and neuron fiber measured in the XY view and XZ view are shown below the corresponding intensity profiles. Scale bar, 50 μm (a) and 5 μm (b).


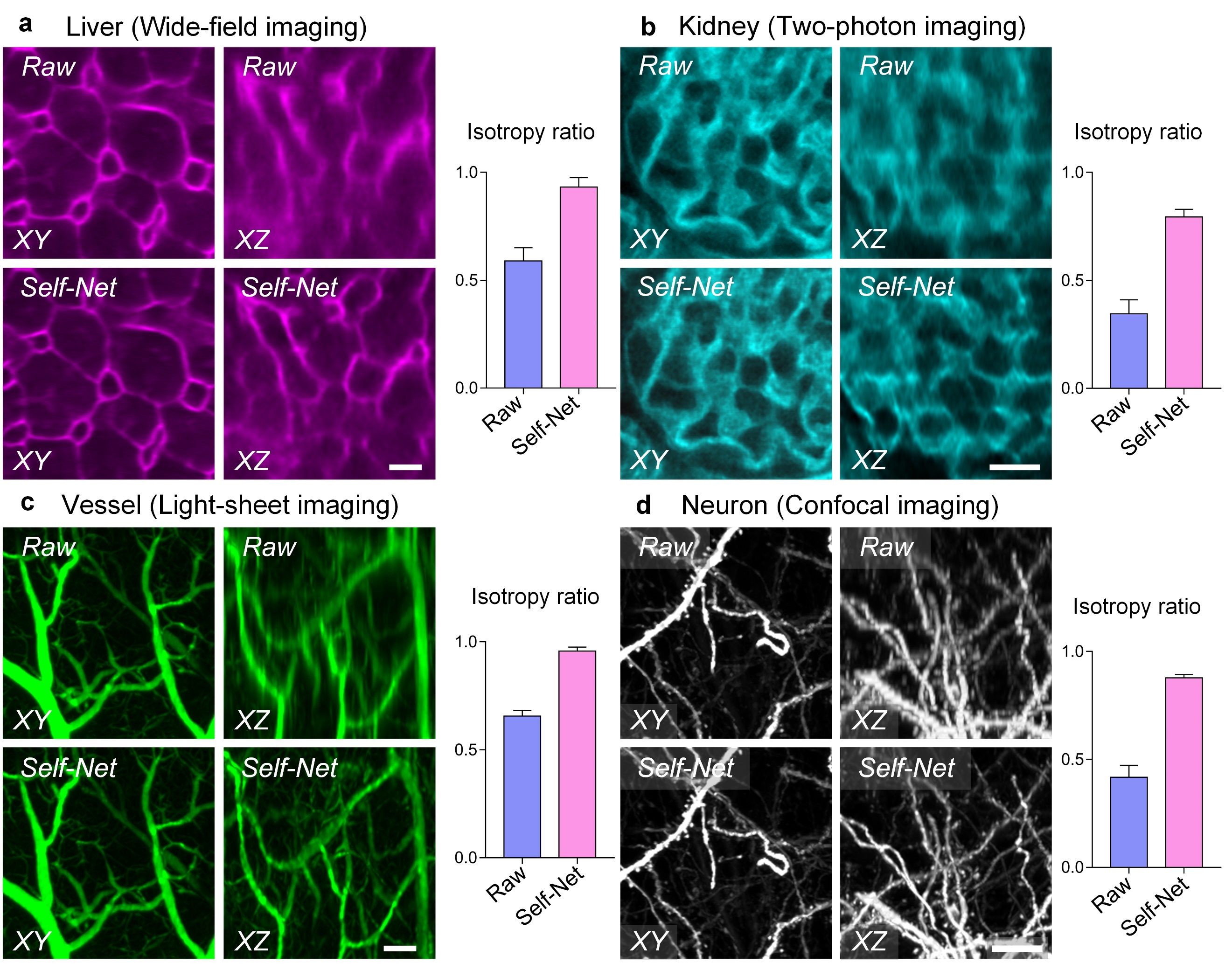


**Fig. S6 Quantifying the isotropy improvement of Self-Net on imaging data of various biological samples and imaging modalities.** As demonstrated in Fig. S5, based on the 3D continuity of the biological structures, for a given 3D image stack, its lateral and axial planes can both capture the same biological structures. Thus, the isotropy ratio can be calculated as the ratio of two FWHM values of the same structure measured in two orthogonal planes (e.g. XY and XZ) of the same 3D image stack (FWHM_XY_/FWHM_XZ_). a-b, Wide-field and two-photon imaging of cleared mTmG mouse liver (a) and kidney tissues (b). The XY and XZ slice of the raw image stack and the Self-Net output is shown on the left. The calculated isotropy ratios are shown on the right (n= 5 blindly chosen tubular structures). Scale bar, 10 μm. c-d, Light-sheet and confocal imaging of cleared mouse brain vasculature (c) and neurons (d), respectively. The XY and XZ MIP of the raw image stack and the Self-Net output is shown on the left. The calculated isotropy ratios are shown on the right. Scale bar, 50 μm (c) and 10 μm (d).


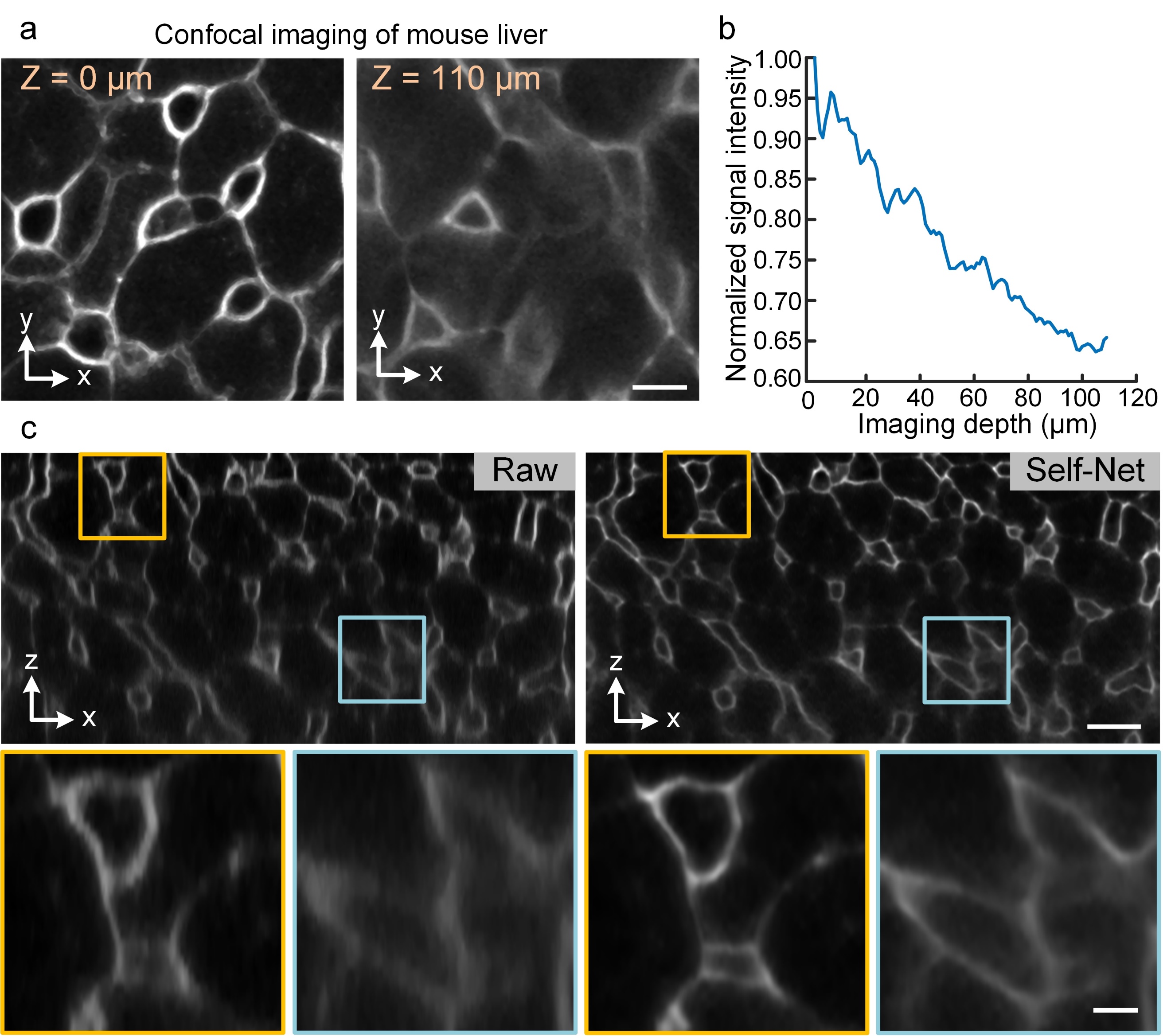


**Fig. S7 Application of Self-Net on imaging data with axial signal attenuation and non-uniform blurring.** A 200-μm thickness cleared liver slice (from a mTmG mouse) was imaged using a Nikon Ni-E A1 confocal microscope with a 60× /1.2-NA water-immersion objective at a voxel size of 0.21 × 0.21 × 1 μm^3^. **a**, Demonstration of two lateral images at different imaging depths. From the visual comparison, it is obvious that the signal intensities and imaging quality of deep images are lower than those of surface images. Scale bar: 10 μm. **b**, Normalized signal intensity as a function of imaging depth. The signal intensity at the imaging depth of 100 μm attenuated to 65% of the signal intensity at the surface. **c**, XZ slice of the raw input and Self-Net output. Two areas in different imaging depths are demonstrated and compared. The results show that Self-Net can improve the resolution isotropy for imaging data with signal attenuation and non-uniform blurring in depth. Scale bar: 20 μm; 5 μm for the enlarged images.


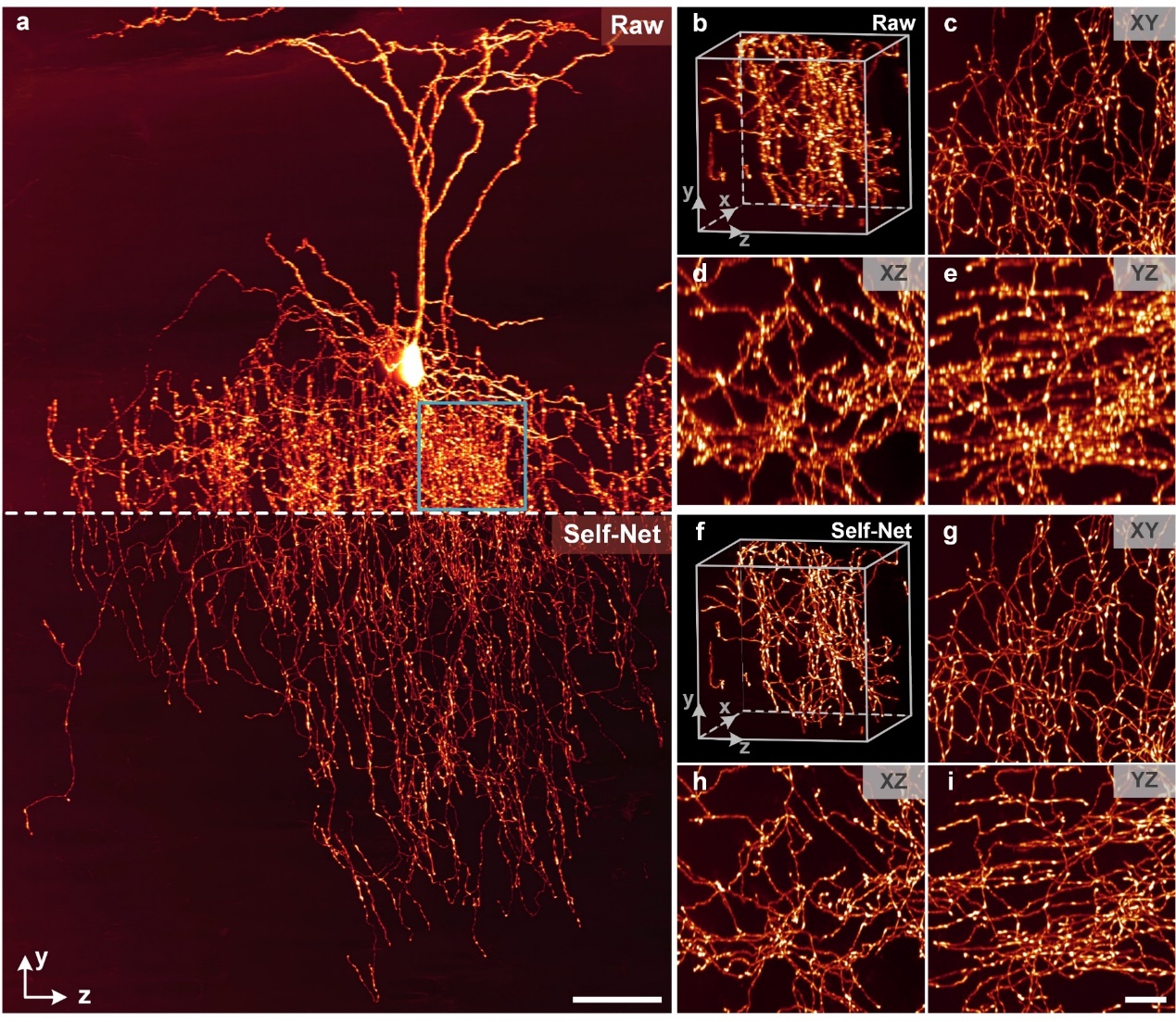


**Fig. S8 Isotropic restoration of an image stack containing a complete axo-axonic cell (AAC). a**, YZ MIP of the whole image volume (296 × 384 × 501 μm^3^). Raw data (top) and Self-Net restoration (bottom) are shown for comparison. Scale bar, 50 μm. **b-i**, 3D reconstruction (b, f), XY MIPs (c, g), XZ MIPs (d, h), and YZ MIPs (e, i) of the data cube (60 × 60 × 60 μm^3^) indicated by the blue box in a. Scale bar, 10 μm.


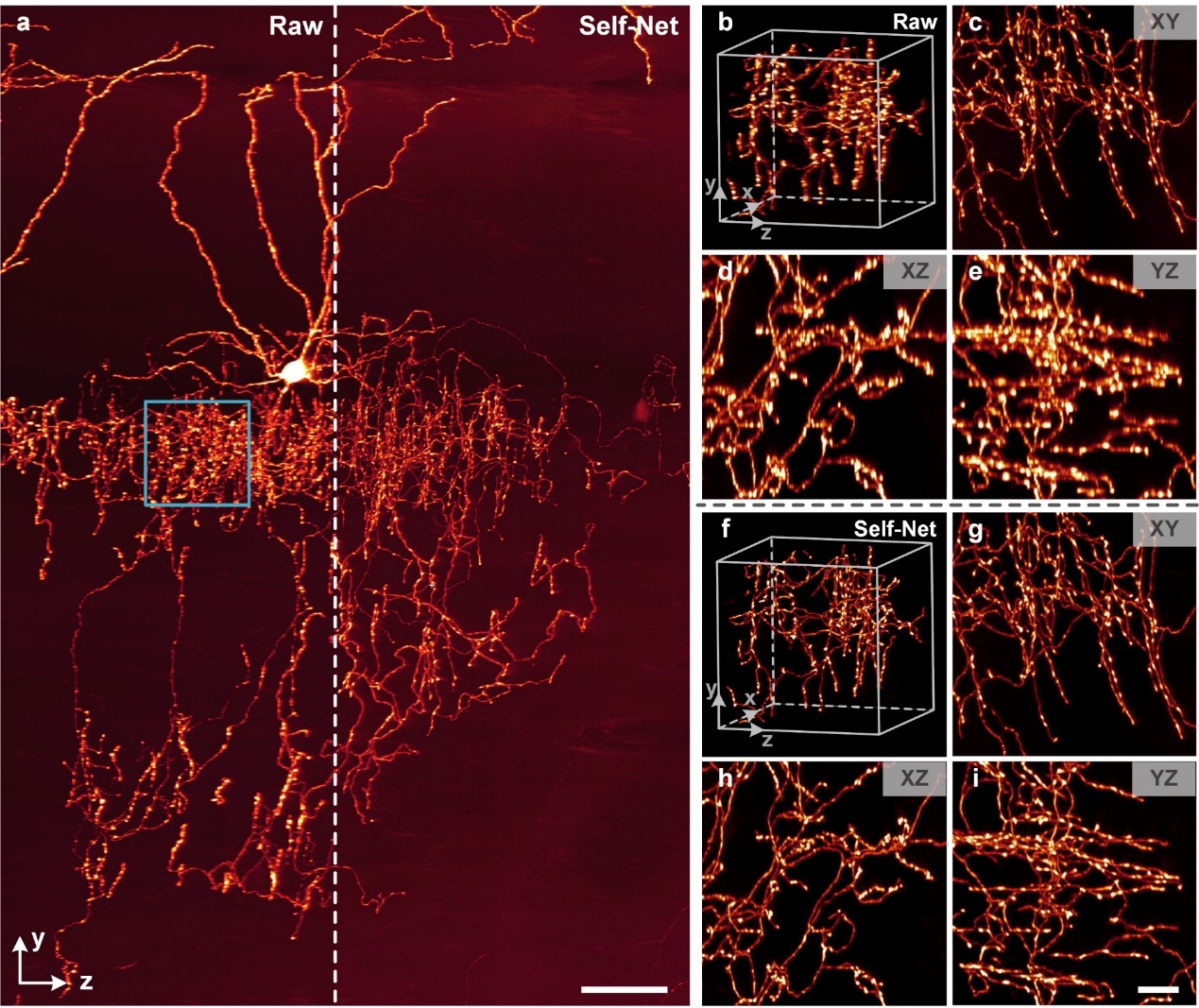


**Fig. S9 Isotropic restoration of another representative AAC image. a**, YZ MIP of the whole image volume (230 × 470 × 400 μm^3^). Raw data (left) and Self-Net restoration (right) are shown for comparison. Scale bar, 50 μm. **b-i**, 3D reconstruction (b, f), XY MIPs (c, g), XZ MIPs (d, h), and YZ MIPs (e, i) of the data cube (60 × 60 × 60 μm^3^) indicated by the blue box in a. Scale bar, 10 μm.


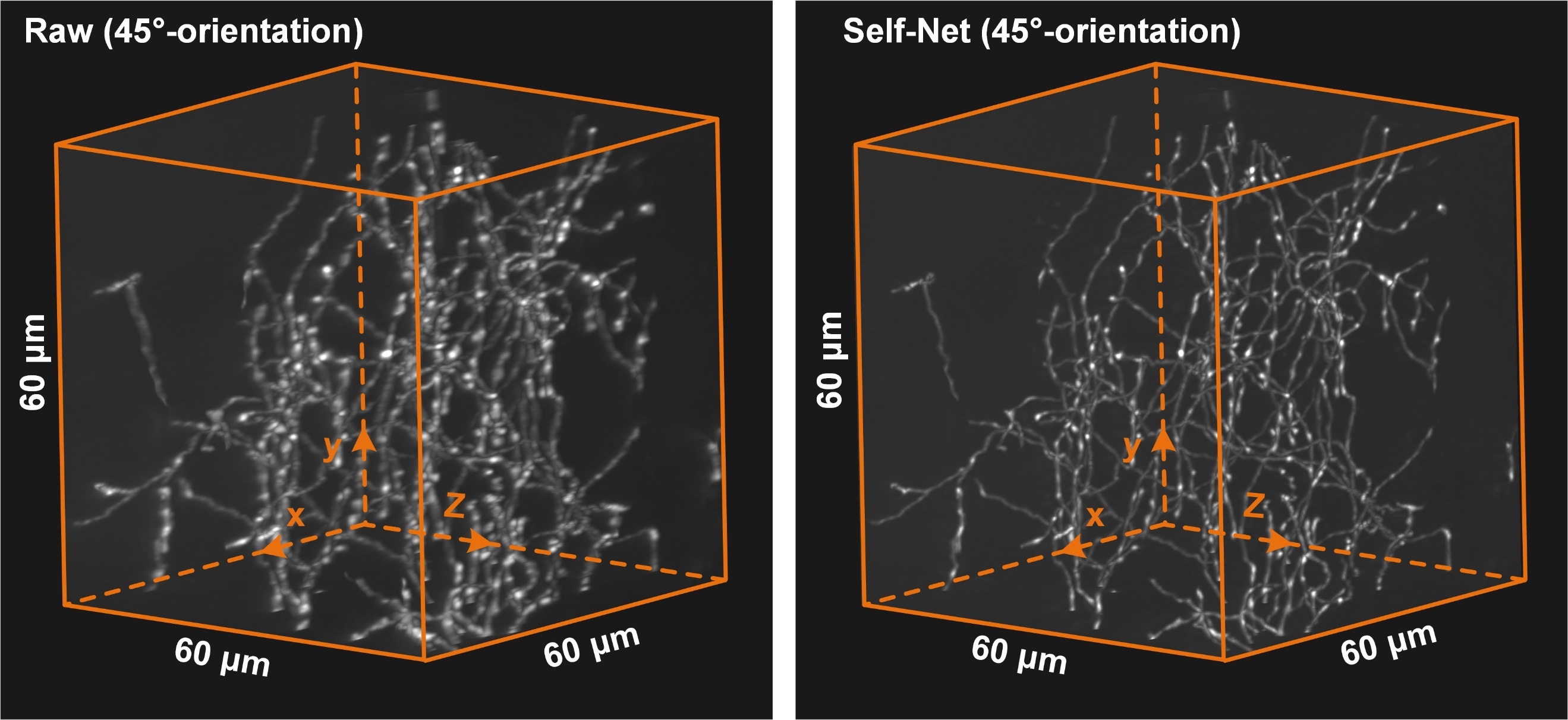


**Fig. S10 Demonstration that the Self-Net reconstructed volume also exhibits enhanced resolution in other directions (such as 45 degrees).**


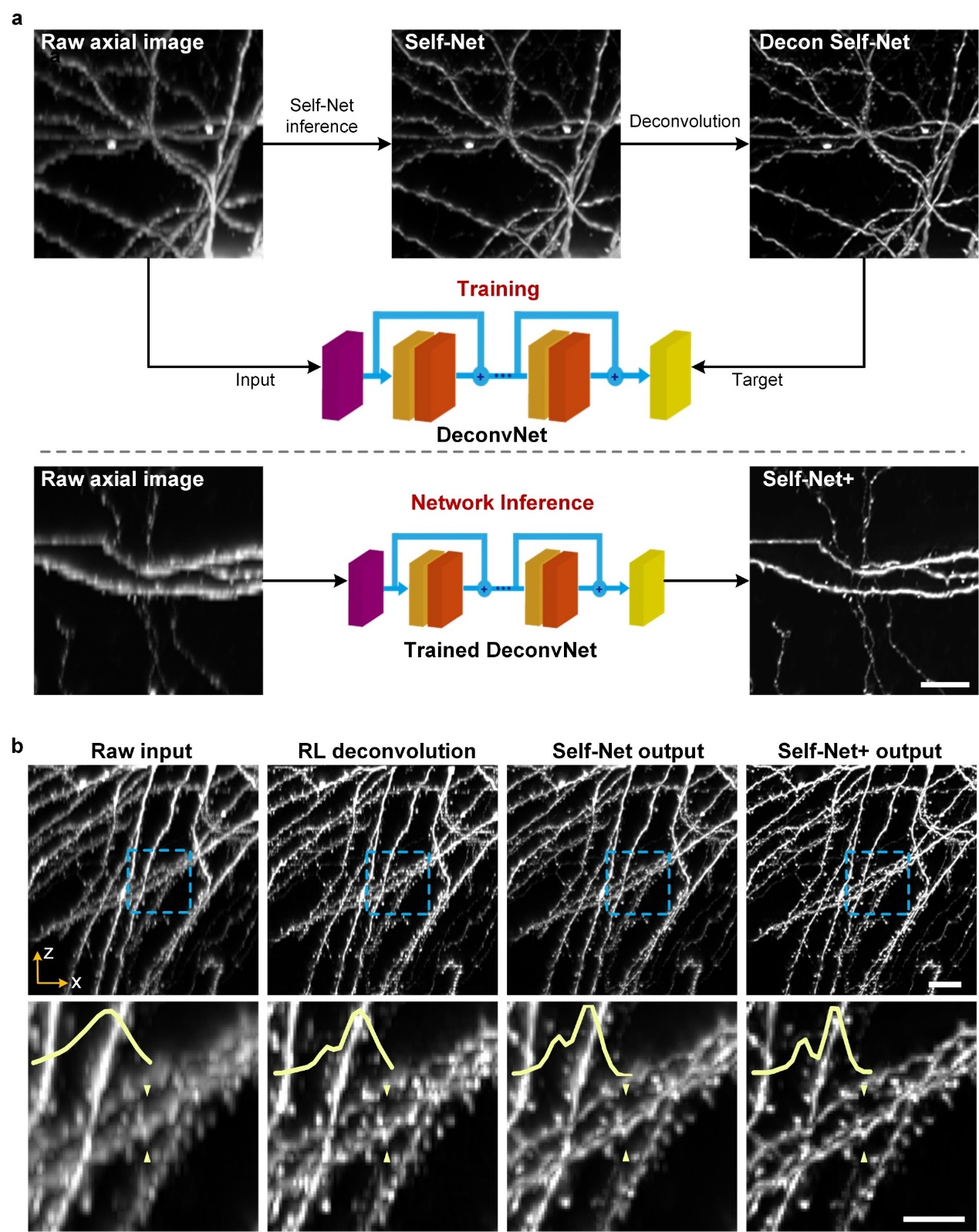


**Fig. S11 Self-Net+, a deconvolution-enhanced Self-Net. a**, Training and testing of Self-Net+. Scale bar, 10 μm. **b**, Comparison of the image restoration performance across the different methods. First row (from left to right): XZ MIPs of the raw data, RL deconvolution, Self-Net output, and Self-Net+ output. The projection thickness is 70 μm. Scale bar, 10 μm. Second row: enlarged views of the dashed box in the first row. Top left: intensity profiles along the lines indicated by the yellow arrowheads. Scale bar, 5 μm.


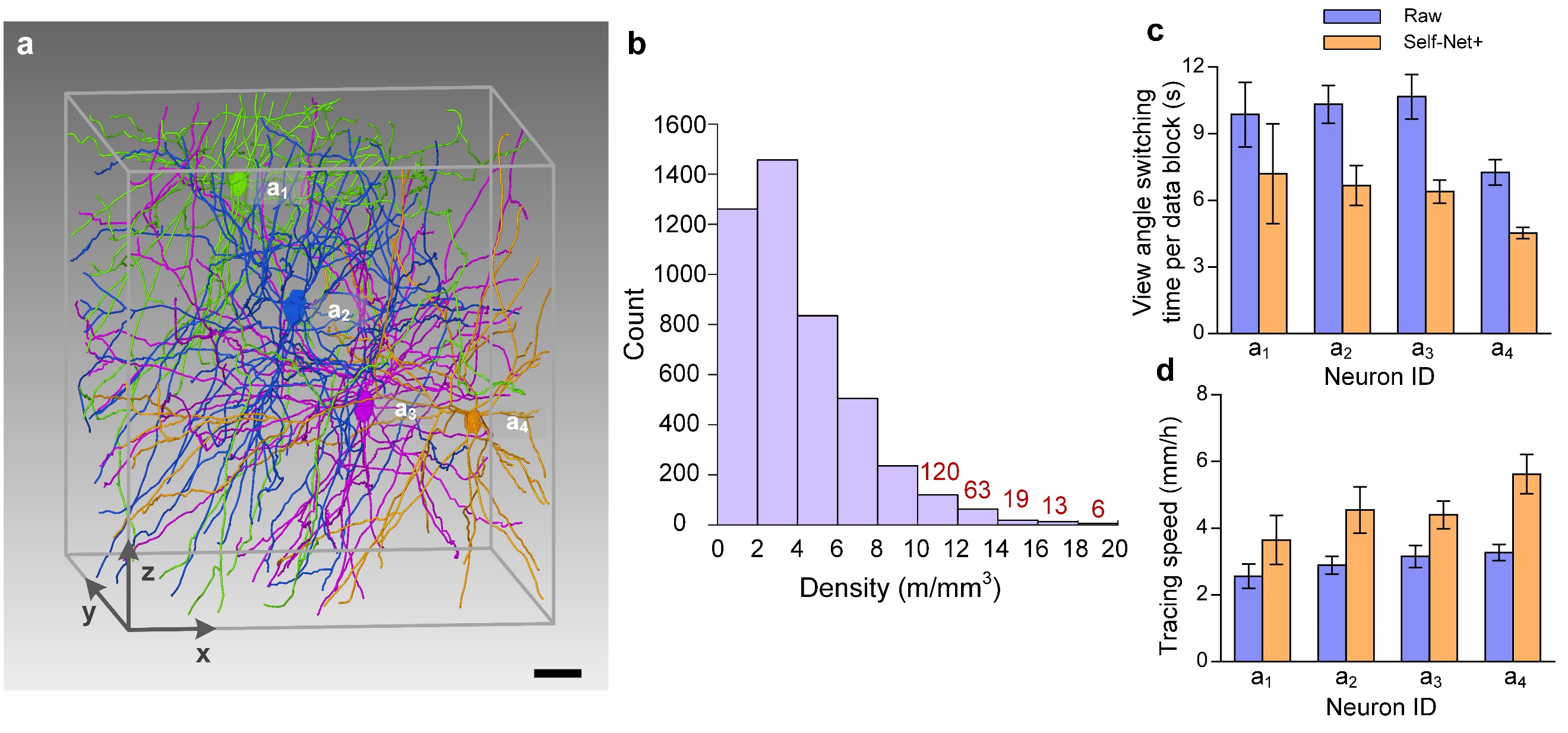


**Fig. S12 Self-Net+ facilitates the neural tracing of dense neuron populations. a**, Gold-standard morphology reconstruction results for the image stack of dense neuron clusters shown in Fig. 4i. Scale bar, 20 μm. The GT reconstruction is the consensus among three neuroanatomical experts independently tracing the same image stack using the raw data. **b**, Neurite density distribution for the data shown in a. The density is calculated by dividing the total length of neuron fibers in the subblock by the volume size (100^3^ voxels). The result shows that highly dense areas with a neurite density ranging from 10 to 20 m/mm^3^ abound in the raw data, revealing the complexity of the data. **c-d**, The viewing angle switching time (c) and reconstruction speed (d) for each neuron in the raw and Self-Net+ data (n = 3 annotators).


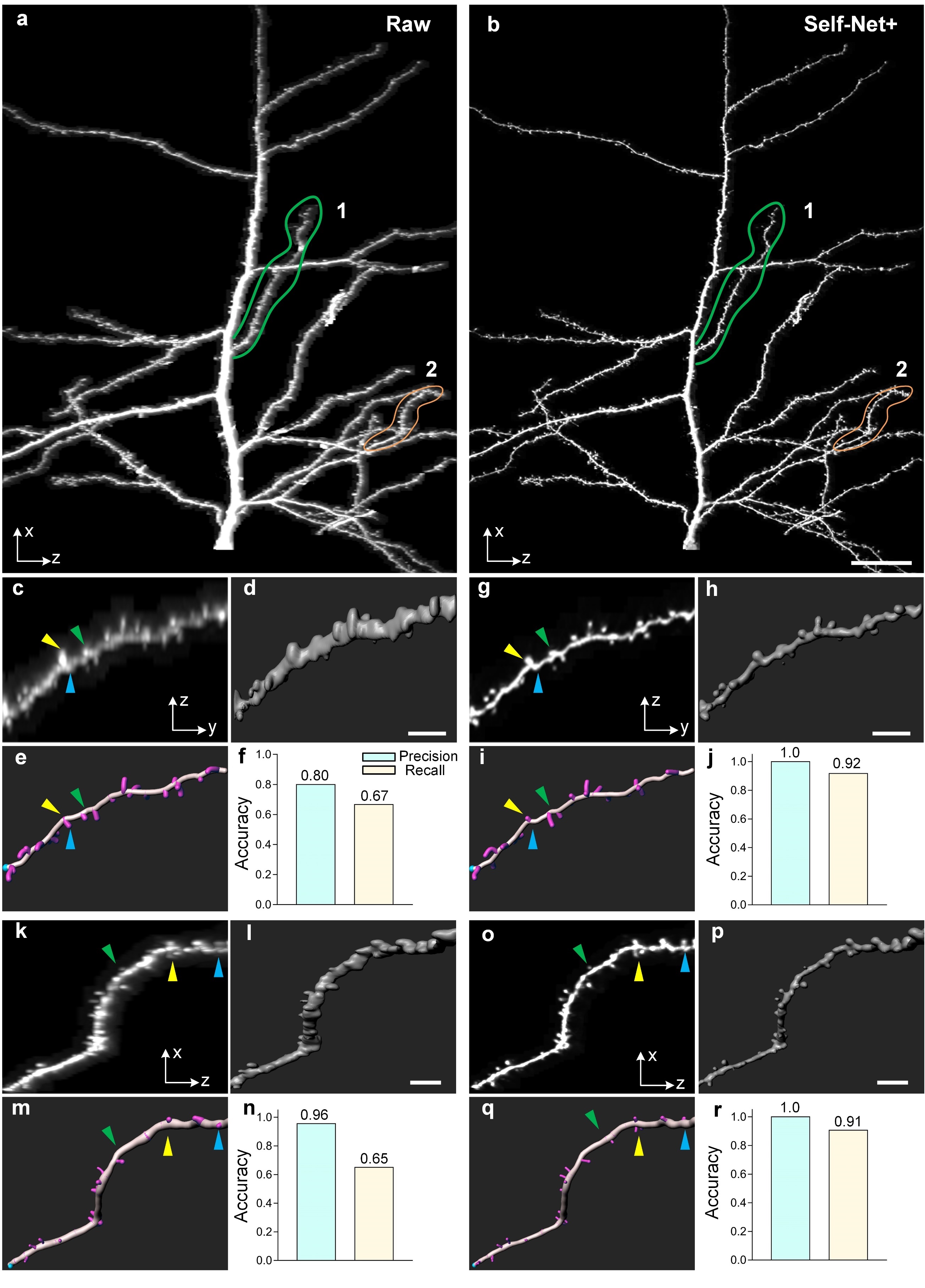


**Fig. S13 Isotropic restoration via Self-Net+ improves 3D observation and automatic detection of dendrite spines. a-b**, XZ MIPs of the raw (a) and Self-Net+ output (b) data (235 × 188 × 363 μm^3^) containing pyramidal neuron dendrites. Scale bar, 25 μm. **c-r**, Automatic dendrite spine detection based on Imaris using the raw (c-f, k-n) and Self-Net+ (g-j, o-r) data, where c-i refer to branch 1 and k-r refer to branch 2. The original MIP images (c, g, k, o), automatic segmentation results (d, h, l, p), spine detection results (e, i, m, q), and statistics of the accuracy (f, j, n, r) are shown. The GT detections of the dendrite spines were derived from the consensus among three neuroanatomical experts, who independently performed detection through slice-by-slice editing in the HR lateral views of the raw data. The yellow arrows highlight the typical spines missing in the raw data but correctly detected in the Self-Net+ data. The blue arrows indicate the erroneously detected spines in the raw data. The green arrows indicate the typical spines that are missing in both the raw and Self-Net+ output data. Scale bar, 5 μm.


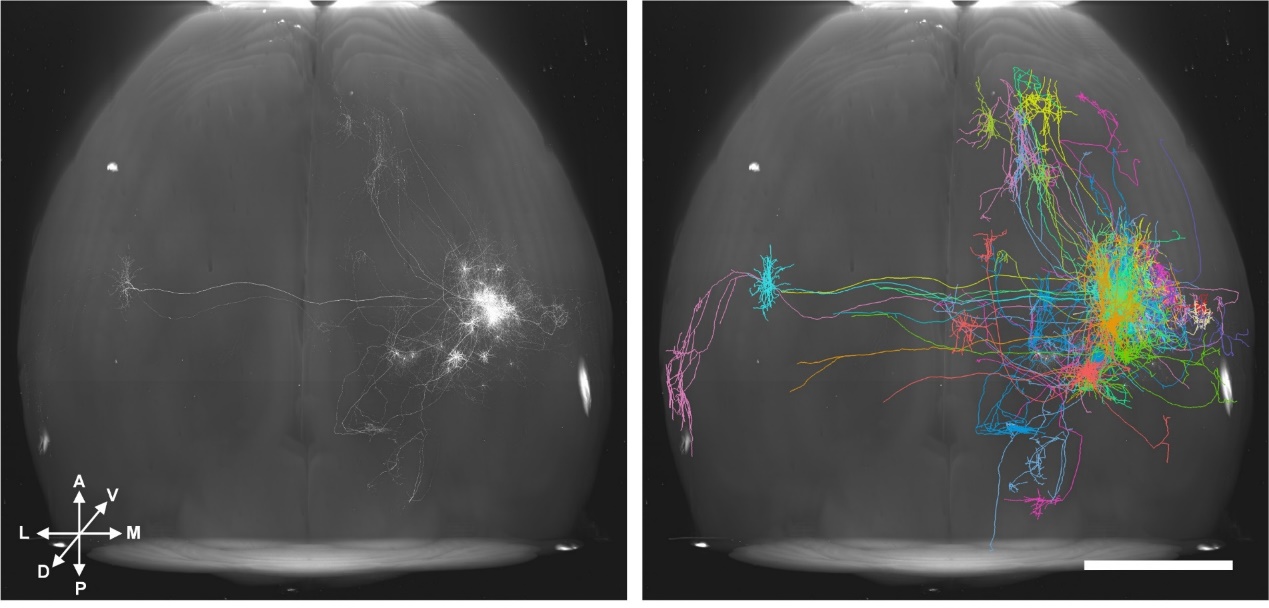


**Fig. S14 Brain-wide neuron morphology reconstruction.** Whole-brain MIP (left) and morphology reconstruction results of 20 long-projection neurons (right). Scale bar, 2 mm.


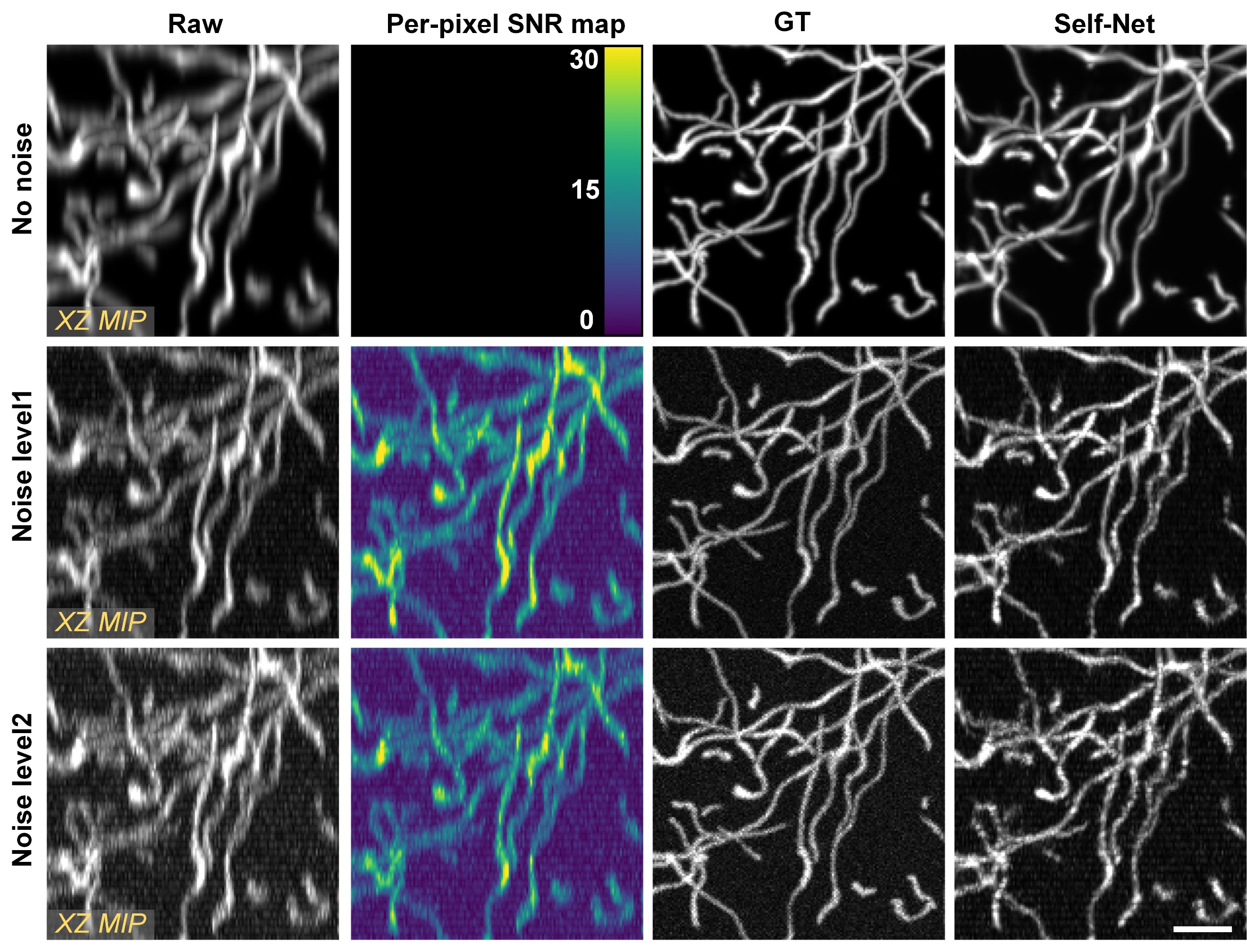


**Fig. S15 Investigating the performance of Self-Net under increased noise.** Rows (from top to bottom): results obtained under different extents of the noise input. The simulated tubular data is the same as used in Fig. 1d. We added different levels of mixed Gaussian-Poisson noise to the clean raw data to simulate low SNR data. Noise level 1: Gaussian noise (std=0.025) and Poisson noise (SNR=20). Noise level 2: Gaussian noise (std=0.025) and Poisson noise (SNR=10). Columns (from left to right): XZ MIP of the raw input, per-pixel SNR map of the raw input, GT, and Self-Net output. The projection thickness is 30 slices. For calculating the per-pixel SNR map, we selected an area of pure background and calculated the mean *b* and standard deviation *σ_b_* of the background. Then the SNR for each pixel was calculated as SNR(*x*,*y*)=|[*s*(*x*,*y*)-*b*]/ *σ_b_* |, where *s* is the pixel value. Scale bar, 30 pixels.


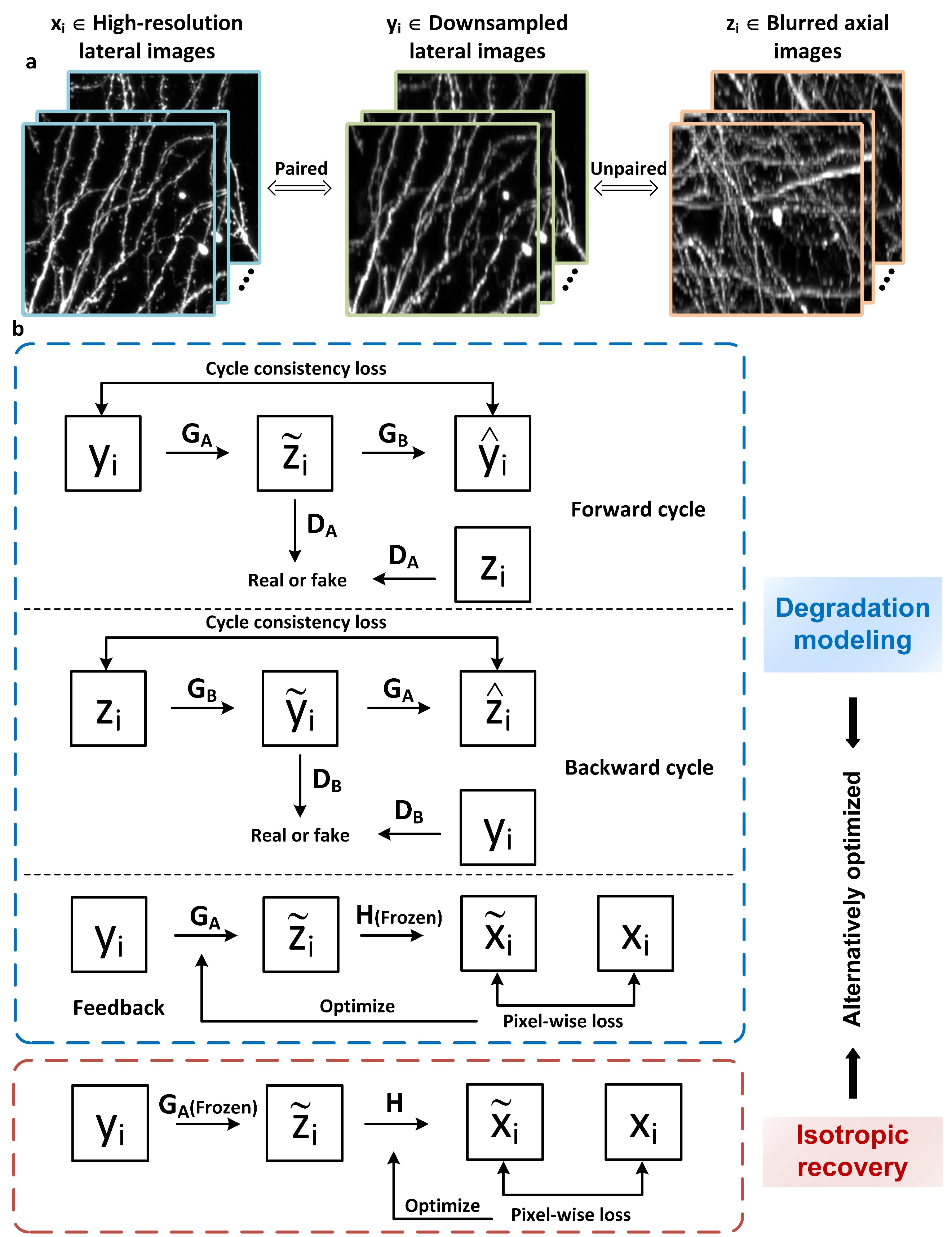


**Fig. S16 Training of Self-Net. a**, Dataset for Self-Net training. **b**, Flowchart of Self-Net training. G_A_ and G_B_ are generators, D_A_ and D_B_ are discriminators, and H represents DeblurNet.


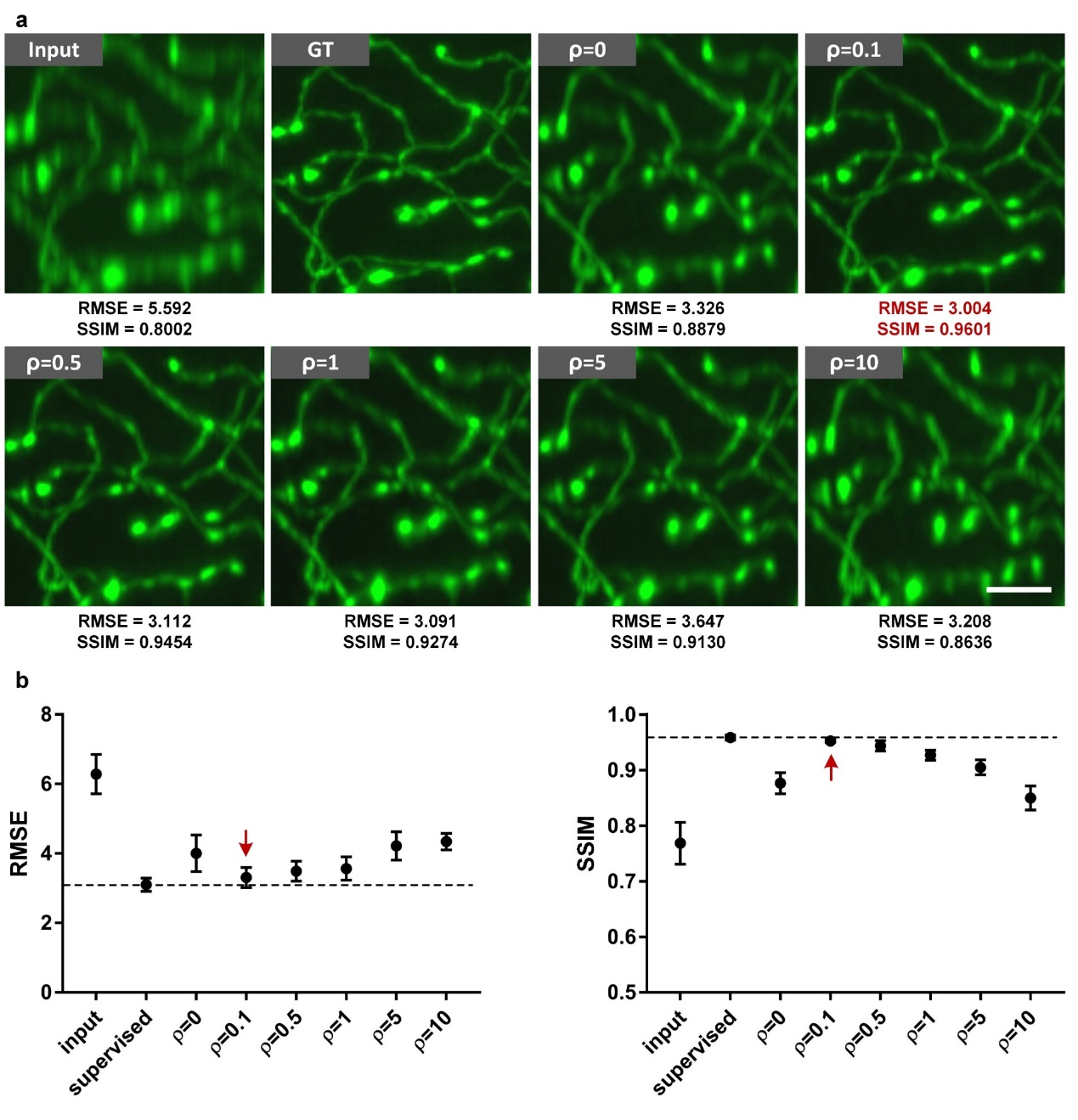


**Fig. S17 Evaluation of the performance of Self-Net under different feedback loss weights. a**, Restoration results obtained with the different weights ρ. Scale bar, 5 μm. **b**, RMSE and SSIM quality metrics (n=5 independent images). The red arrows highlight the results (ρ=0.1) closest to the supervised training results.


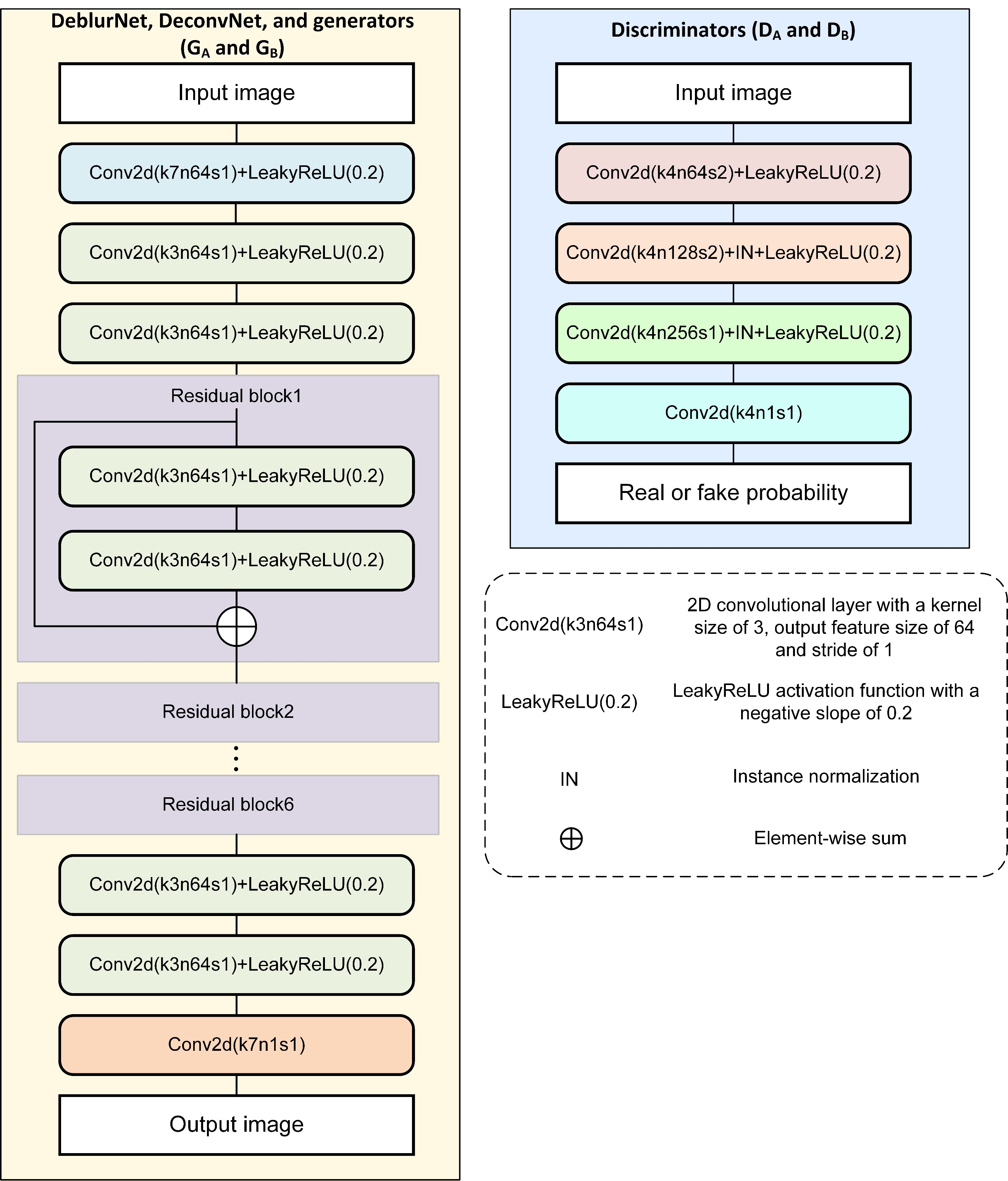


**Fig. S18 Network architecture of Self-Net.** DeblurNet, DeconvNet and the generators are constructed with residual blocks, and the discriminators are based on PatchGAN.


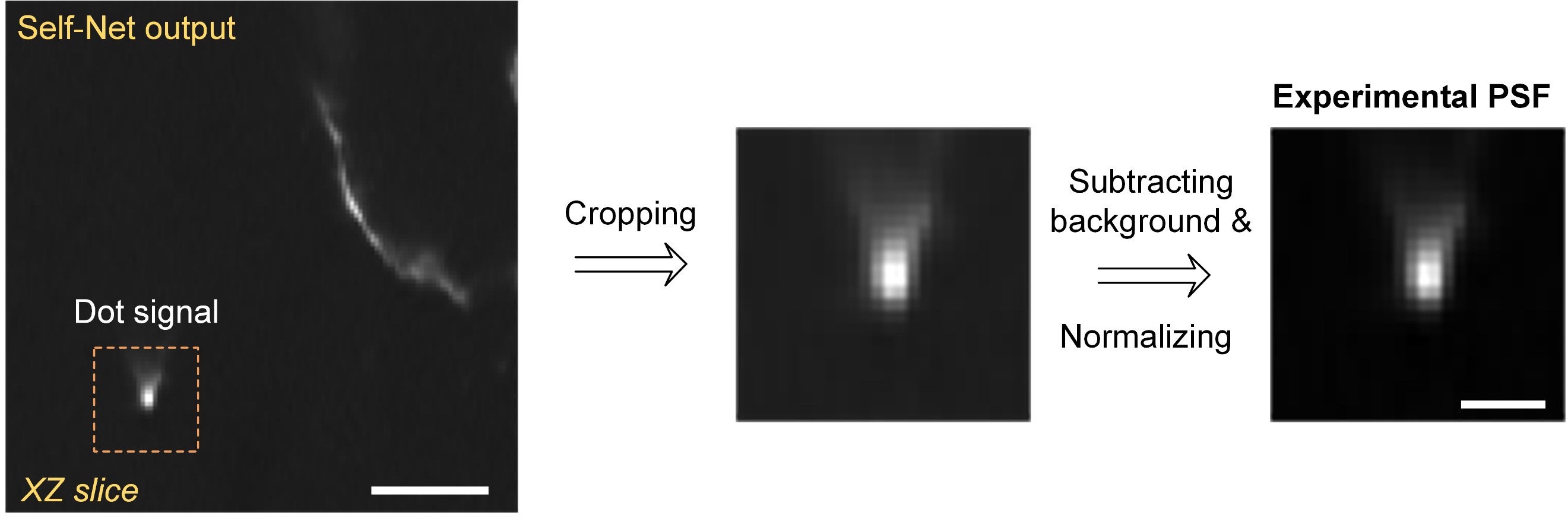


**Fig. S19 Demonstration of extracting experimental PSF for the deconvolution process in the Self-Net+ method.** An isolated fluorescent dot signal was cropped from the XZ slice of the Self-Net output. The cropped image patch had a size of 50 × 50 pixels and was centered on the dot signal. After subtracting the background and normalizing, the image can be served as the PSF kernel for RL deconvolution. Scale bar, 10 μm; 5 μm for the enlarged images.

**Supplementary Tables**

**Supplementary Table 1|Detailed information on the training data used in this work.** X: high-resolution lateral images, Y: down-sampled lateral images, Z: blurred axial images. The volume size W x H x D represent voxels in x, y, and z dimensions, N is the number of volumes.

|  | Volume size  (W × H × D × N) | Training data size  (Number of image patches) | | |
| --- | --- | --- | --- | --- |
|  |  | X | Y | Z |
| Simulated fluorescent beads (Fig. 1b) | 512 × 512 × 512 × 1 | 6404 | 6404 | 6380 |
| Simulated tubular (Fig. 1d) | 1024 × 1024 × 1024 × 1 | 4216 | 4216 | 4135 |
| Semi-synthetically generated data (Fig. 1g) | 2298 × 1060 × 237 × 1 | 4221 | 4221 | 4221 |
| Mouse liver (Fig. 2a) | 1800 × 1700 × 246 × 1 | 4274 | 4274 | 4337 |
| Mouse kidney (Fig. 2b) | 1024 × 1024 × 150 × 1 | 4800 | 4800 | 4080 |
| Mouse brain vessel (Fig. 2c) | 916 × 916 × 498 × 1 | 4585 | 4585 | 4728 |
| Mouse brain neurons  (Fig. 2d) | 1024 × 1024 × 90 × 2 | 4715 | 4715 | 4897 |
| Fluorescent nanobeads  (Fig. 3a) | 305 × 305 × 30 × 7 | 1699 | 1699 | 1657 |
| Mitochondria (Fig. 3d) | 1024× 1024 × 32× 7 | 4210 | 4210 | 3612 |
| Axo-axonic cell  (AAC, Fig. 4a) | 2046× 1140 × 551× 1 | 4897 | 4897 | 4674 |
| Dense neuron cluster  (Fig. 4i) | 1153× 893 × 194× 1 | 2791 | 2791 | 2436 |

**Supplementary Table 2|Acquisition parameters and approximate isotropy ratios (see Methods for more details, mean ± std, N=5) for real data shown in this work.**

|  | Imaging system | Objective | Voxel size | Isotropy ratio of raw data |
| --- | --- | --- | --- | --- |
| Mouse liver (Fig. 2a) | Wide-field | 20× 1.0 NA | 0.32 × 0.32 × 1 μm^3^ | 0.59 ± 0.06 |
| Mouse kidney  (Fig. 2b) | Two-photon | 60× 1.2 NA | 0.21 × 0.21 × 1 μm^3^ | 0.35 ± 0.06 |
| Mouse brain vessel (Fig. 2c) | Light-sheet | 4× 0.28 NA | 1.63 × 1.63 × 3 μm^3^ | 0.65 ± 0.03 |
| Mouse brain neurons (Fig. 2d) | Confocal | 60× 1.2 NA | 0.21 × 0.21 × 1 μm^3^ | 0.42 ± 0.05 |
| Fluorescent nanobeads  (Fig. 3a) | STED | 60× 1.4 NA | 15 × 15 × 50 nm^3^ | 0.37 ± 0.03 |
| Mitochondria  (Fig. 3d) | Instant SIM | 60× 1.45 NA | 55.5 × 55.5 × 250 nm^3^ | 0.56 ± 0.02 |
| Axo-axonic cell  (AAC, Fig. 4a) | CS-fMOST | 60× 1.0 NA | 0.2 × 0.2 × 1 μm^3^ | 0.51 ± 0.10 |
| Dense neuron cluster (Fig. 4i) | CS-fMOST | 60× 1.0 NA | 0.2 × 0.2 × 1 μm^3^ | 0.50 ± 0.03 |

**Supplementary Table 3|Comparison of the reconstruction speed of AAC neurons using the raw and Self-Net data.** Three AACs in both the raw and Self-Net data were traced separately by an experienced annotator. The tracing interval in different data exceeds more than one week to eliminate corresponding memory.

| **Neuron ID** | **Morphology parameters** | | | **Morphology reconstruction** | | | | | |
| --- | --- | --- | --- | --- | --- | --- | --- | --- | --- |
|  | **Length (mm)** | **Branches** | **Maximum**  **branch order** | **Time (h)** | | **Speed (μm/h)** | | **Average**  **speed (μm/h)** | |
|  |  |  |  | **Raw** | **Self-Net** | **Raw** | **Self-Net** | **Raw** | **Self-Net** |
| 01 | 13.8 | 791 | 35 | 23.1 | 6.2 | 597 | 2226 | 602 | 2378 (395%) |
| 02 | 16.5 | 1113 | 29 | 29.6 | 7.3 | 557 | 2260 |  |  |
| 03 | 32.1 | 1775 | 36 | 49.3 | 12.1 | 651 | 2653 |  |  |

**Supplementary Table 4|Testing isotropic recovery performances of online training Self-Net and Self-Net+ on randomly-selected five CS-fMOST whole-brain imaging systems.** These systems were equipped with 0.8 NA water immersion objectives. Five mice brains with different strains and virus labeling strategies were imaged separately by these systems at a voxel size of 0.35 × 0.5 ×1 μm^3^. The isotropy ratio of the raw, Self-Net output, and Self-Net+ output of each whole-brain data was quantified using the same method described in Fig. S5 (n= 5 randomly selected fibers).

|  | Sample information | Isotropy ratio | | |
| --- | --- | --- | --- | --- |
|  |  | Raw | Self-Net | **Self-Net+** |
| 01 | C57BL/6J mouse with sparse virus labeling | 0.57 ± 0.06 | 0.95 ± 0.06 | **0.97 ± 0.03** |
| 02 | C57BL/6J mouse with sparse virus labeling | 0.48 ± 0.10 | 0.90 ± 0.05 | **0.98 ± 0.01** |
| 03 | C57BL/6J mouse with sparse virus labeling | 0.44 ± 0.07 | 0.90 ± 0.06 | **0.96 ± 0.02** |
| 04 | Batf3-IRES2-FlpO mouse with sparse virus labeling | 0.51 ± 0.04 | 0.92 ± 0.07 | **0.98 ± 0.03** |
| 05 | Rxfp1-P2A-Cre mouse with sparse virus labeling | 0.47 ± 0.04 | 0.88 ± 0.06 | **0.97± 0.04** |

**Supplementary Table 5|Decision accuracy of local complex areas using the raw and isotropic data and negative impacts of local misidentifications on the reconstruction of long-range projection neurons.** The statistical results are based on three experienced annotators, who independently traced 100 data blocks in 3D in the test dataset (randomly-shuffled raw and Self-Net outputs of 50 local complex regions). The tracing GT for the test set was derived from the reconstruction consensus among three neuroanatomical experts, who independently traced the raw data through slice-by-slice manual editing in the HR lateral views.

| Data | Annotator ID | Decision accuracy of local complex areas  (n = 50) | | | Negative impacts of local misidentifications on the reconstruction of long-range projection neurons | |
| --- | --- | --- | --- | --- | --- | --- |
|  |  | Right | Wrong | Error rate | Accumulated erroneously-traced length (mm) | Accumulated erroneously-traced branches |
| Raw | 01 | 30 | 20 | 40% | 38.6 | 519 |
|  | 02 | 32 | 18 | 36% | 32.9 | 440 |
|  | 03 | 33 | 17 | 34% | 40.4 | 563 |
|  | Average ± SD | 32 ± 1.5 | **18 ± 1.5** | **36.7±3.0%** | **37.3 ± 3.9** | **507 ± 62** |
| Self-Net | 01 | 49 | 1 | 2% | 0.6 | 7 |
|  | 02 | 50 | 0 | 0% | 0 | 0 |
|  | 03 | 48 | 2 | 4% | 4.6 | 81 |
|  | Average ± SD | 49 ± 1 | **1 ± 1** | **2±2%** | **1.7 ± 2.5** | **29 ± 45** |

**Supplementary video captions**

**Supplementary video 1, 3D visualizations of the raw anisotropic data, isotropic ground truth (GT) data, OT-CycleGAN, and Self-Net restoration of simulated beads.** See also **Fig. 1b**.

**Supplementary video 2, Self-Net enables isotropic 3D imaging of diverse biological samples acquired by different microscopies.** Isotropic restoration of volume acquisitions of mTmG mouse liver and kidney is shown. The liver data was acquired with a wide-field microscope (NA 1.0) at 0.32 × 0.32 × 1 μm^3^ voxel size. The kidney data was acquired with a two-photon microscope (NA 1.2) at 0.21 × 0.21 × 1 μm^3^ voxel size. The video shows continuous axial (xz) slices of the raw image stack and Self-Net output. The video also demonstrated isotropic restoration of volume acquisitions of mouse brain vessels and neurons. The vessel data was acquired with a light-sheet microscope (NA 0.28) at 1.63 × 1.63 ×3 μm^3^ voxel size. The Thy1-mouse brain neuron data were acquired with a confocal microscope (NA 1.2) at 0.21 × 0.21 × 1 μm^3^ voxel size. 3D volume rendering of the raw image stack and Self-Net output is shown in the video. See also **Fig. 2**.

**Supplementary video 3, 3D visualizations of the Z-STED, partial Z-STED data, and Self-Net output of fluorescent nanobeads.** The Z-STED data was acquired using 100% depletion power for Z at 30 × 30 × 30 nm^3^ voxel size. The partial Z-STED data was acquired using 30% depletion power for Z and 70% depletion power for XY at 15 × 15 × 50 nm^3^ voxel size. Self-Net was trained and deployed on the partial Z-STED data for isotropic restoration and to push the resolution limit of 3D STED. See also **Fig. 3a**.

**Supplementary video 4, Axial views of raw mitochondrial data acquired by iSIM and the corresponding Self-Net restorations.** Fixed U2OS cells transfected with mEmerald-Tomm20 labeling outer mitochondrial membrane were acquired with the iSIM super-resolution system at 55 × 55 × 250 nm^3^ voxel size. The video shows continuous axial (xz) slices of the raw image stack and Self-Net output. The yellow arrows highlight the mitochondrial membrane blurred in the raw axial views was super-resolved after Self-Net isotropic restoration. See also **Fig. 3d**.

**Supplementary video 5, 3D visualizations of the raw anisotropic data and Self-Net restorations of the AAC neuron.** 3D raw image stack (220 × 400 ×550 μm^3^) of the AAC neuron was acquired by a CS-fMOST whole-brain imaging system at 0.2 × 0.2 × 1 μm^3^. See also **Fig. 4a**.

**Supplementary video 6, Self-Net+ restorations of a dense neuron cluster at the injection site of a sparsely labeled whole-brain imaging dataset.** The raw data was acquired by a CS-fMOST whole-brain imaging system at 0.2 × 0.2 × 1 μm^3^. See also **Fig. 4i**.

**Supplementary video 7, Demonstration of a typical reconstruction error (proceeding along an incorrect branch) in neuron morphology reconstruction using the raw anisotropic data.** The raw data was acquired by a CS-fMOST whole-brain imaging system at 0.2 × 0.2 × 1 μm^3^. After Self-Net+ restoration of the same area, the neurites became clear in all perspectives and the annotators can readily determine the correct proceeding direction. The 3D tracing results using the Self-Net+ data were consistent with GT. The tracing GT was derived from the reconstruction consensus among three neuroanatomical experts, who independently performed tracing through slice-by-slice editing in the HR lateral views of the raw data. See also **Fig. 4m**.

**Supplementary video 8, Demonstration of another typical reconstruction error (missing branch) in neuron morphology reconstruction using the raw anisotropic data.** See also **Fig. 4n**.

**Supplementary video 9, Demonstration of on-demand isotropic restoration in whole-brain neuron reconstruction.** The video shows the annotators traversing data cubes in the whole-brain data along the extension direction of the neurites. When encountering challenging areas, they can perform real-time isotropic restoration on these areas for better observation and tracing. While for other easy areas, they can directly trace in raw data. See also **Fig. 5b**.

**Supplementary video 10, Demonstration of typical challenging local areas in whole-brain imaging data.** The raw data was acquired by a CS-fMOST whole-brain imaging system at 0.2 × 0.2 × 1 μm^3^. 3D volume rendering of the raw image stack and Self-Net output is shown in the video.

**Supplementary video 11, Demonstration of a local misidentification using the raw anisotropic data can lead to severe reconstruction error for a long-range projection neuron.** The raw data was acquired by a CS-fMOST whole-brain imaging system at 0.2 × 0.2 × 1 μm^3^. After isotropic restoration, the annotators could easily identify the correct proceeding direction of each neurite, thus reducing the occurrence of severe reconstruction errors. See also **Fig. 5g**.
